# A wearable biosensing platform for continuous monitoring of inflammatory and metabolic biomarkers for real‐time health tracking and personalized care

**DOI:** 10.1002/btm2.70104

**Published:** 2026-01-16

**Authors:** Annapoorna Ramasubramanya, Preeti Singh, Akash Kumar, Kai‐Chun Lin, Shalini Prasad, Sriram Muthukumar

**Affiliations:** ^1^ Department of Bioengineering University of Texas at Dallas Richardson Texas USA; ^2^ EnLiSense LLC Allen Texas USA

**Keywords:** circadian biomarkers, cortisol, IL‐6, melatonin, stress physiology, sweat biosensing, TNF‐α

## Abstract

Wearable biosensors have the potential to revolutionize health monitoring, yet reliable, time‐resolved hormone and cytokine tracking remains elusive. This study introduces a dual‐framework approach to enable circadian and immune profiling through perspired sweat sensing. First, sweat–saliva thresholds were calibrated for cortisol, melatonin, interleukin‐6 and tumor necrosis factor‐alpha, achieving significant classification performance (Area Under the Curve >0.80) for physiologically relevant salivary benchmarks. Second, circadian rhythmicity of each biomarker was modeled using circaCompare, revealing distinct oscillatory patterns stratified by age, gender, and stress. Young adults exhibited robust melatonin–cortisol phase separation and rhythmic immune signals. Older participants showed dampened amplitudes, phase shifts, and inflammatory dominance. Notably, stress exposure induced earlier cortisol peaks (Δ ≈ 6.7 h), suppressed melatonin rhythms, and heightened immune amplitude variability—hallmarks of circadian misalignment. These findings establish sweat as a valid, real‐time medium for capturing endocrine and immune cycles, with analytical tools capable of uncovering early physiological strain. This work lays a foundation for personalized chronobiological monitoring and stress‐risk screening in naturalistic settings.

**Translational Impact Statement**

This work establishes perspired sweat as a clinically meaningful, non‐invasive fluid for continuous circadian and immune monitoring. By linking sweat biomarker levels to validated salivary thresholds and modeling their rhythms across age, gender, and stress, this study enables passive, personalized tracking of physiological resilience. The analytical approach introduced here provides a foundation for intelligent biosensor algorithms that can detect hormonal misalignment, immune activation, or stress‐induced circadian disruption—supporting early intervention, behavior adjustment, and tailored health strategies in both every day and clinical settings.

## 1 INTRODUCTION

Circadian rhythms orchestrate a wide range of physiological processes—including hormone secretion, immune modulation, and behavioral regulation—with disruptions linked to numerous chronic conditions such as depression, metabolic syndrome, and cardiovascular disease.^1^, ^2^ Among key molecular markers of circadian integrity, melatonin and cortisol serve as well‐established indicators of central clock timing and hypothalamic–pituitary–adrenal (HPA) axis activity, respectively.^3^ Additionally, pro‐inflammatory cytokines such as interleukin‐6 (IL‐6) and tumor necrosis factor‐alpha (TNF‐α) are tightly regulated by peripheral circadian clocks and stress‐reactivity pathways, bridging endocrine and inflammatory axes.^4^, ^5^

Despite growing recognition of these interactions, studying them in real‐world settings remains challenging. Conventional sampling methods—saliva, serum, or urine—are episodic, often invasive, and poorly suited for capturing dynamic temporal variation. Longitudinal sampling is logistically complex, especially outside clinical environments, and often lacks the resolution needed to disentangle circadian misalignment from acute or chronic physiological strain. Furthermore, population‐level variability in age, sex, and stress responses introduces heterogeneity that traditional approaches rarely stratify or resolve.

In contrast, passive sweat biosensing offers a promising non‐invasive modality for continuous physiological monitoring via skin‐interfaced wearables.^6^, ^7^, ^8^ However, the field currently lacks robust analytical frameworks capable of translating sweat biomarker concentrations into clinically meaningful metrics or dynamic rhythmic profiles. Few studies have established validated equivalency thresholds between sweat and salivary markers or examined whether sweat can resolve biologically relevant variation across demographic and psychological dimensions.^9^, ^10^, ^11^

To address these limitations, we developed a dual‐analytical framework that^1^ derives sweat–saliva equivalency thresholds for circadian and inflammatory biomarkers, and^2^ applies stratified 24‐h harmonic curve fitting using the circaCompare model. This approach moves beyond static concentration snapshots to enable personalized, time‐resolved biomarker interpretation.

We report that passive sweat sensing not only mirrors salivary biomarker classification for cortisol, melatonin, IL‐6, and TNF‐α, but also captures distinct circadian signatures stratified by age, sex, and psychological stress. By integrating dynamic chronobiological modeling with physiological subgroup analysis, this study introduces a scalable method for real‐world circadian phenotyping—advancing wearable biosensing from signal detection to decision support.

Beyond its biomarker scope, the novelty of this study lies in its analytical convergence: combining validated sweat–saliva equivalency thresholds with circadian harmonic modeling across endocrine and immune axes in diverse populations. Unlike prior approaches that treat sweat concentrations as static proxies, this framework reveals dynamic rhythmic architecture—stratified by age, sex, and stress exposure—while maintaining clinical interpretability. By unlocking the temporal dimension of passive biosensing, this method introduces a personalized chronobiology toolkit that can detect subclinical misalignment, guide behavioral interventions, and inform closed‐loop wearable systems. In doing so, it repositions sweat analytics not merely as a passive stream, but as a rhythm‐sensitive lens for proactive health surveillance—offering time‐aware diagnostics for a precision wellness future.

**FIGURE 1:**
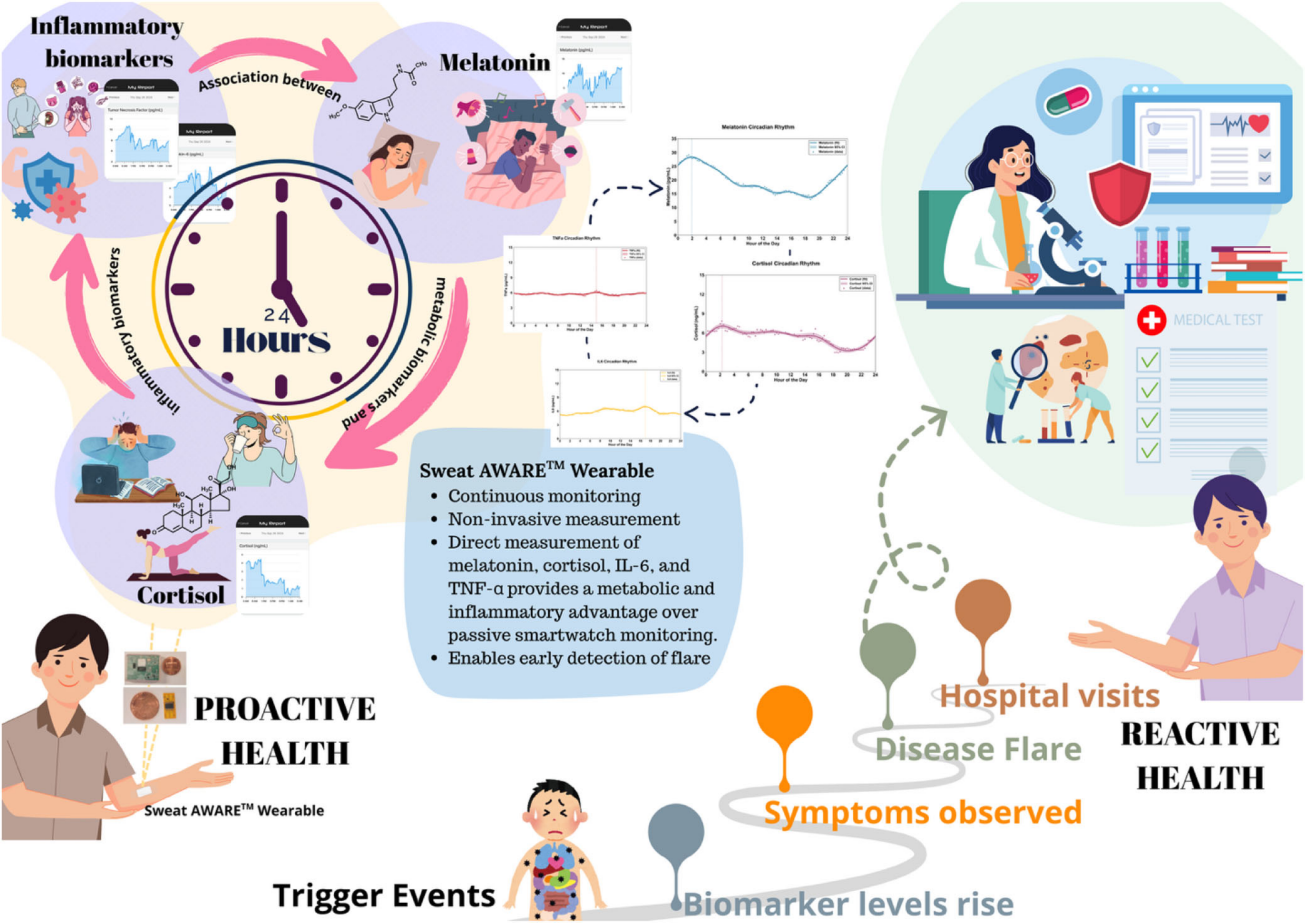
Integration of passive sweat biosensing into proactive health monitoring frameworks. The schematic contrasts reactive health models—which rely on symptom emergence and delayed biomarker assessment—with a proactive approach enabled by the Sweat AWARE™ wearable. Continuous, non‐invasive measurement of melatonin, cortisol, IL‐6, and TNF‐α allows dynamic tracking of endocrine and inflammatory activity across the day. This capability supports early identification of physiological dysregulation, potentially preventing disease flare‐ups and reducing hospital visits. Red signal curves depict acute inflammatory responses captured in real time, while shaded circadian bands illustrate temporal windows of biomarker fluctuation. Together, this visualization positions sweat analytics as a metabolic and immunological advantage over conventional passive smartwatch monitoring. IL‐6, interleukin‐6; TNF‐α, tumor necrosis factor‐alpha.

This translational framework is visually represented in Figure 1, which contrasts reactive, symptom‐driven health systems with a proactive biosensing paradigm enabled by continuous sweat monitoring. The Sweat AWARE™ platform exemplifies this shift by enabling non‐invasive tracking of circadian (melatonin, cortisol) and immune (IL‐6, TNF‐α) biomarkers. Through early detection of physiological dysregulation—prior to clinical onset—this strategy supports personalized, preventive care models that are scalable and adaptable to populations at risk for stress‐related, metabolic, or inflammatory disorders.

## 2 MATERIALS AND METHODS

### 2.1 Sensor and sensing system

The passive sweat biosensing system consists of a skin‐interfaced perspiration biosensor integrated with a compact electronic reader worn on the forearm, secured with a medical‐grade adhesive patch (Figure 1). This wearable platform enables continuous, non‐invasive monitoring by wirelessly transmitting impedance‐based biochemical data via Bluetooth to a paired mobile application. The app relays these encrypted data streams to a secure cloud environment for further processing and biomarker classification.

The electronic reader is engineered with an Advanced Reduced Instruction Set Computer Machine (ARM) Cortex core processor, a battery management unit, and onboard temperature and humidity sensors to ensure contextual tracking of dermal conditions. The reader is powered by a rechargeable lithium‐polymer battery and features a Bluetooth Low Energy (BLE) module to facilitate power‐efficient, real‐time communication.^12^ This configuration supports dynamic assessment of sweat biomarker concentrations while simultaneously logging environmental parameters such as skin temperature and moisture—critical for interpreting sensor output under varying physiological conditions. This integrated hardware–software system forms a resilient foundation for passive biosensing, elevating sweat‐based analytics into a scalable modality for real‐time health tracking beyond traditional clinical environments.

#### 2.1.1 Sensor functionalization and characterization

The aptamers and antibodies were employed to functionalize the sensor platform, enabling high‐affinity and target‐specific detection of cortisol, melatonin, IL‐6, and TNF‐α. Initially, a cortisol‐specific 5′‐thiol‐modified aptamer was freshly prepared and functionalized on top of the designated sensor channel. The aptamer self‐assembled onto the ZnO surface via thiol‐ZnO interactions, forming a stable self‐assembled monolayer. Sensors were incubated at 4°C for a few hours, followed by lyophilization and vacuum sealing storage. The melatonin detection was achieved using a melatonin‐specific monoclonal antibody.^13^ The antibody was conjugated with DTSSP by NHS‐ester mediated crosslinking. Then the solution was functionalized on the sensor channel and incubated at 4°C, then vacuum sealed and lyophilized. For the inflammation detection, the IL‐6 and TNF‐α specific 5′‐thiol‐modified aptamer was drop‐cast onto the sensor channels. The thiol‐metal binding created a homogenous SAM on the ZnO surface. After the aptamer loading, the sensors were maintained at 4°C for incubation, then lyophilized and sealed.^14^ We lyophilized the sensors to extend the shelf life of the sensors.

To confirm surface functionalization, Nicolet iS50 Fourier‐transform infrared (FTIR) spectroscopy was used to verify the presence of aptamer and antibody—DTSSP conjugates on the ZnO‐coated substrate, scanning over a spectral range of 4000–400 cm^−1^. Bruker atomic force microscopy (AFM) analysis revealed nanoscale surface morphology, while scanning electron microscopy illustrated the highly porous architecture of the ZnO layer (Figure S5a), favorable for analyte diffusion and enhanced sensor responsiveness. Elemental composition was confirmed via energy‐dispersive x‐ray spectroscopy (Figure S5b), which showed dominant Zn and O signals—consistent with ZnO deposition—and minor Ag peaks, indicative of the electrode structure. Together, these characterization methods validated the structural integrity and functional readiness of the sensor platform.

### 2.2 Transformation to concentration levels using regression models

The dataset included 43 participants, split into training (*n* = 31) and test (*n* = 12) sets. To transform raw signals from the sweat biosensor into biomarker concentration values, we developed new machine learning models for cortisol and melatonin, while adapting existing validated models for IL‐6 and TNF‐α.^15^, ^16^ Saliva values were interpolated to create a continuous target variable. Multiple regression models were evaluated using *R* ^2^, RMSE, and concentration range. CatBoost Regressor demonstrated the best performance—yielding *R* ^2^ scores of 0.984 (cortisol) and 0.955 (melatonin)—with no negative predictions (Table S8). XGBoost also performed well (*R* ^2^ = 0.968 for cortisol, 0.956 for melatonin), while models like Support Vector Regression showed low predictive power and higher error, reflecting limited suitability for the dataset's complexity.^13^

### 2.3 Human subject recruitment for passive sweat biosensing system testing

The passive sweat biosensing system consisted of a perspiration biosensor affixed to an electronic reader worn on the forearm using a medical‐grade adhesive patch (Figure 1a). The reader—featuring an ARM core processor, temperature and humidity sensors, and a battery management system—transmitted impedance data to a mobile application via BLE, enabling real‐time monitoring and secure cloud storage.^12^

Perspiration data were collected from 43 healthy participants (*n* = 20 female, *n* = 23 male) across three age groups: <25, 25–40, and >40 years (Table S9). The study received IRB approval (IRB 24‐2, University of Texas at Dallas), and informed consent was obtained. Saliva samples were collected at 12 discrete time points over 48 h—initial setup began at 16:00, followed by collections at 19:00, 21:00, 22:00, 23:00 (T1–T4), then 07:00, 08:00 (T5–T6), again at 19:00, 21:00, 22:00, 23:00 (T7–T10), and concluding with T11 and T12 at 07:00 and 08:00 the following day (Figure 1b). This schedule was designed to capture diurnal fluctuations in cortisol and melatonin.

Before device attachment, forearms were prepped with alcohol wipes, and lyophilized biosensors were activated using a wet wipe. Devices remained in place for 48 h, secured with SkinGrip patches. The biosensor captured real‐time perspiration and temperature data, while participants used the mobile app to log sleep patterns, mood, saliva collection times, medications, and dietary intake.^13^

## 3 RESULTS

### 3.1 Characterization of the modified sensing platform

The sweat sensor platform employed a zinc oxide (ZnO) thin film as the electrode interface for immobilizing aptamers and antibodies, enabling targeted biosensing. Surface characterization using attenuated total reflectance FTIR (ATR‐FTIR) spectroscopy and AFM was conducted to validate probe chemisorption on the ZnO substrate.

Figure S1a illustrates ATR‐FTIR spectra for three sensor states: unmodified ZnO, DTSSP–antibody conjugate, and thiolated aptamer‐functionalized ZnO nanosheets. The unmodified ZnO exhibited a distinct peak at 868 cm^−1^, corresponding to characteristic Zn–O lattice vibrations below 1000 cm^−1^ .^17^ Surface functionalization introduced several additional vibrational features. A prominent peak at 989 cm^−1^ was associated with epoxy groups from the aptamer sequence, while a band at 1082 cm^−1^ corresponded to phosphate group stretching within the nucleic acid backbone—both confirming aptamer immobilization.^18^

The DTSSP crosslinker, used for antibody attachment, generated an NHS ester‐associated peak at 1640 cm^−1^. Upon enzyme conjugation, cleavage of the C—O bond within the NHS ester caused a measurable reduction in peak intensity, as shown in Figure S1b.^19^ Additionally, a 1648 cm^−1^ peak in the aptamer‐modified surface was attributed to double bond stretching (C=N, C=O, C=C), indicative of DNA and RNA base stacking interactions.

A weak absorption near 2542 cm^−1^—associated with S—H stretching from DTSSP's thiol groups—was absent in pristine ZnO and diminished post‐immobilization, as seen in Figure S1c.^19^, ^20^, ^21^ Collectively, these spectroscopic signatures confirm robust probe anchoring to the ZnO substrate via phosphate coordination and thiol–metal interactions, establishing a stable platform for the selective binding of sweat‐borne analytes.

**FIGURE 2:**
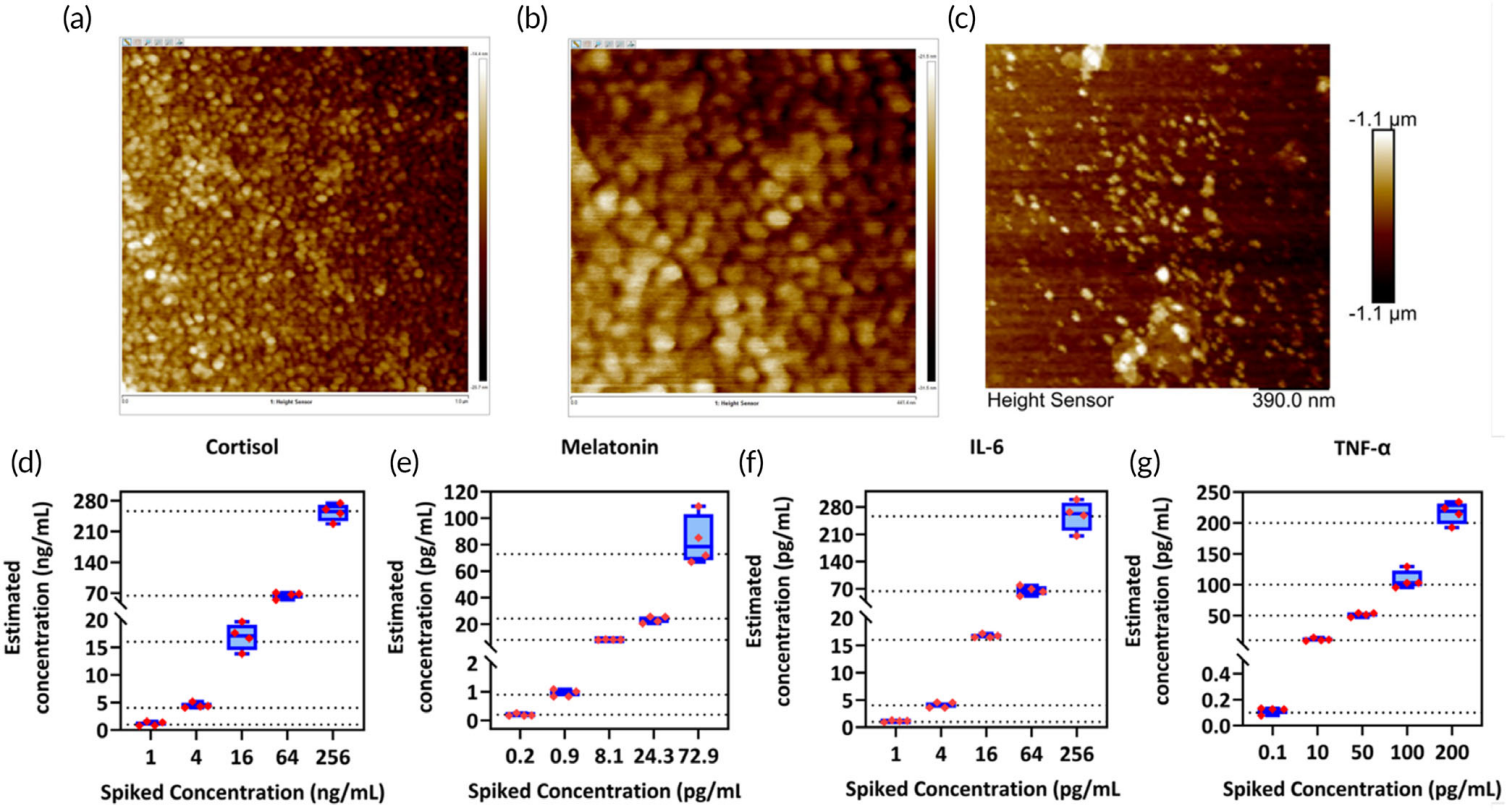
AFM images of only ZnO (a), DTSSP–antibody–ZnO (b), aptamer–ZnO. Box plot (d–g) showing the spiked and their estimated concentrations, indicating the accurate measurement of cortisol (d), melatonin (e), IL‐6 (f), and TNF‐α (g) for *N* = 4 replicates each. AFM, atomic force microscopy; IL‐6, interleukin‐6; TNF‐α, tumor necrosis factor‐alpha.

The surface morphology of the ZnO‐based sweat sensor at various stages of functionalization—bare ZnO, DTSSP–antibody conjugation, and aptamer attachment—was characterized using AFM on ZnO‐coated glass substrates (Figure 2a–c). As shown in Figure 2a, the pristine ZnO surface exhibited a uniform granular texture, with nanoparticle sizes ranging from 15 to 17 nm. Following functionalization with the DTSSP crosslinker and melatonin antibody (Figure 2b), the surface displayed distinct spherical protrusions with an average particle size of approximately 35–40 nm, indicative of successful conjugation and antibody immobilization. In Figure 2c, the aptamer‐functionalized ZnO surface revealed uniformly distributed nanoscale features, with the aptamer layer contributing a height of ~5–10 nm atop the ZnO matrix. Collectively, these AFM observations—alongside corresponding spectral validation—confirm effective and stable incorporation of both antibody and aptamer moieties on the ZnO platform, establishing a reliable biointerface for selective sweat‐based analyte detection.^22^

Figure 2 illustrates the analytical performance of the sweat sensor platform for detecting four key biomarkers: IL‐6, TNF‐α, cortisol, and melatonin. Calibration curves were first established using serial dilutions of spiked standards, followed by concentration recovery validation. Each subplot (Figure 2d–g) presents box plots comparing estimated sensor concentrations to known spiked inputs, highlighting accuracy and consistency.

Figure 2d demonstrates the sensor's ability to detect cortisol across a broad dynamic range (1–256 ng/mL). Minimal data scatter and close alignment of median lines with the reference (dotted) line indicate high fidelity in cortisol quantification—critical for continuous stress monitoring. Table S1 confirms that recovery percentages remained within the CLSI‐accepted range of 80–120%.

Figure 2e shows comparable accuracy for melatonin detection across 0.2–72.9 pg/mL, with consistent signal outputs and recovery percentages again within the 80–120% range (Table S1), supporting potential applications in sleep‐related monitoring.

Figure 2f highlights IL‐6 quantification over a dynamic range of 1–256 pg/mL, with well‐aligned boxplots reflecting accurate detection of inflammatory signals. Table S2 lists corresponding recovery rates, all within acceptable CLSI limits.

Figure 2g presents results for TNF‐α ranging from 0.1 to 200 pg/mL. Low variability and median concordance with spiked concentrations reflect the sensor's sensitivity to early‐stage inflammatory cytokine levels. Recovery values (Table S3) fell within the targeted 80–120% window. All assays were repeated in quadruplicate to confirm reproducibility.

Following quantitative validation, sensor specificity was assessed by exposing each channel to physiologically relevant concentrations of non‐target analytes (Table S4). The cortisol sensor showed cross‐reactivity below 20% when challenged with IL‐6, TNF‐α, and melatonin. The melatonin sensor demonstrated outstanding specificity with <10% cross‐reactivity. IL‐6 channels exhibited minimal interference—0.66%, 3.45%, and 3.54% when exposed to TNF‐α, cortisol, and melatonin, respectively—while TNF‐α sensors maintained <20% response to off‐target inputs.

Collectively, these findings confirm that the sweat sensor platform achieves accurate, specific, and simultaneous detection of multiple biomarkers, supporting its application in non‐invasive, real‐time physiological monitoring (Tables S5–S8).

### 3.2 Stratification and ROC analysis

Following the completion of the sweat–saliva equivalency modeling, circadian sampling protocol, and statistical framework described above, we next evaluated how well passive sweat concentrations reflected salivary biomarker thresholds across physiological and temporal states. The results below highlight the discriminatory capacity of sweat‐derived measurements for circadian hormones and inflammatory cytokines, along with their classification performance using ROC analysis.

Sweat concentrations of cortisol and melatonin demonstrated strong correspondence with clinically validated salivary ranges. Specifically, sweat cortisol values of <2.4, 2.4–7.9, and >7.9 ng/mL aligned with salivary reference intervals of <3, 3–7, and >7 ng/mL, respectively. Similarly, sweat melatonin levels stratified into <6.5, 6.5–42.0, and >42.0 pg/mL, mapping to salivary thresholds of <10, 10–40, and >40 pg/mL. These equivalency bins were derived from simultaneous sample collection events, where each data point represents a paired measurement: the salivary concentration captured at a precise time point, immediately matched with the sweat concentration acquired from the adjacent sensor readout.

**FIGURE 3:**
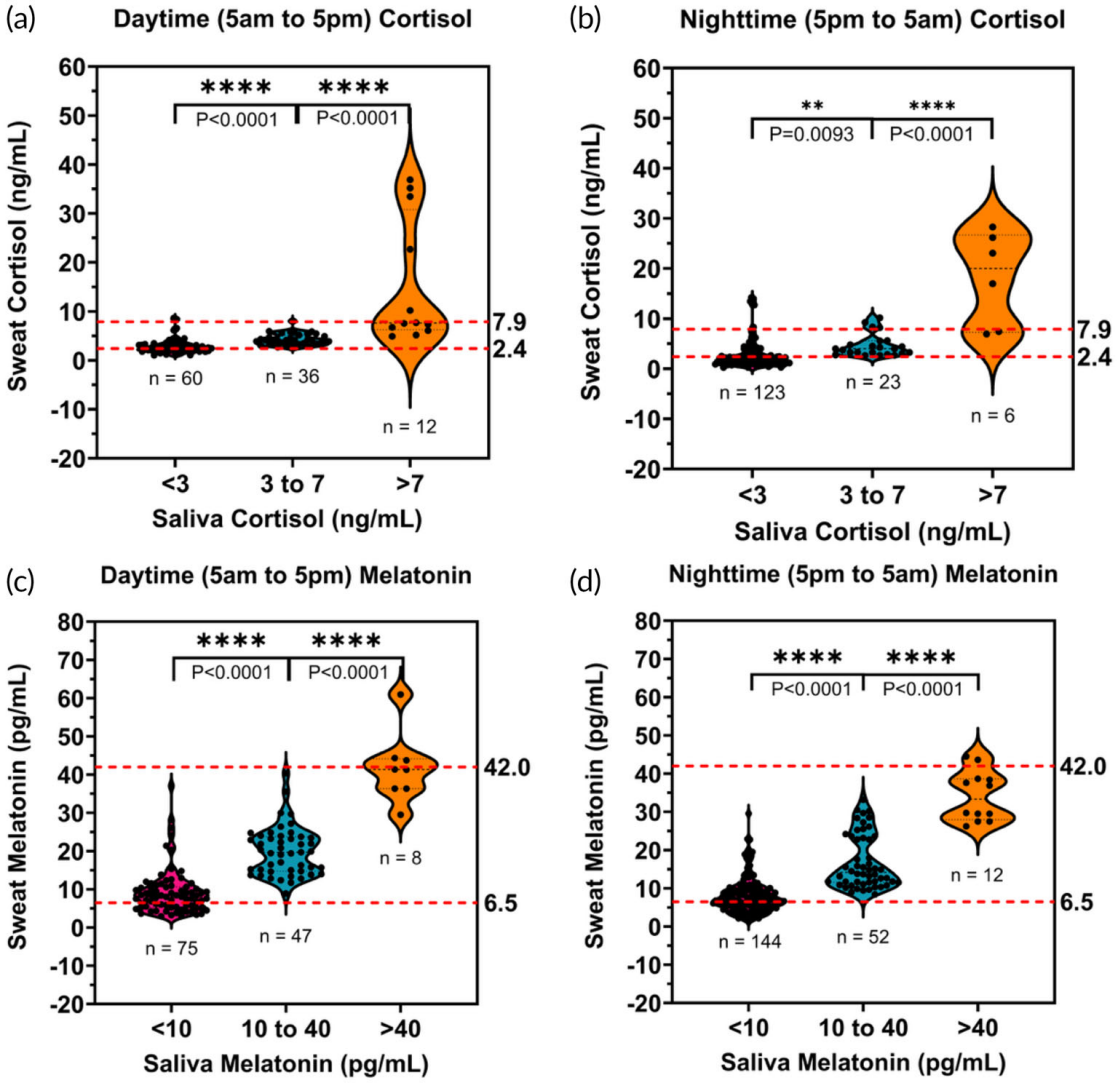
Correlation of sweat and salivary hormone levels across circadian windows. Violin plots display sweat cortisol and melatonin levels stratified by corresponding salivary concentration bins during daytime (5 a.m. to 5 p.m.) and nighttime (5 p.m. to 5 a.m.). (a) Daytime sweat cortisol levels significantly increased across salivary thresholds: <3 ng/mL (*n* = 60), 3–7 ng/mL (*n* = 36), and >7 ng/mL (*n* = 12); *p* < 0.0001. (b) Nighttime sweat cortisol mirrored salivary bins: <3 ng/mL (*n* = 123), 3–7 ng/mL (*n* = 23), and >7 ng/mL (*n* = 6); *p* = 0.0093, *p* < 0.0001. (c) Daytime sweat melatonin aligned with salivary ranges: <10 pg/mL (*n* = 75), 10–40 pg/mL (*n* = 37), >40 pg/mL (*n* = 8); *p* < 0.0001. (d) Nighttime sweat melatonin tracked rising salivary melatonin: <10 pg/mL (*n* = 40), 10–40 pg/mL (*n* = 12), >40 pg/mL (*n* = 12); *p* < 0.0001. Red dashed lines indicate validated sweat threshold bins. These results confirm that sweat accurately reflects salivary hormone levels and preserves circadian variation, with statistical significance across all physiological strata.

Statistically significant distributional differences were observed across bins (*p* < 0.0001) in both daytime and nighttime intervals (Figure 3), affirming the ability of sweat to reliably reflect salivary hormone dynamics. Notably, daytime melatonin levels were not close to zero—neither in saliva nor sweat—despite biological expectations. This is attributable, in part, to basal melatonin tone during the diurnal phase, as well as inherent assay sensitivity: the reference salivary values used to calibrate these bins were derived from a standard ELISA‐based commercial kit, which exhibits non‐zero lower detection limits and physiologically relevant tailing in daytime signal. As such, sweat values replicate this non‐zero background rather than absolute hormone suppression, reflecting both biological baseline and measurement characteristics.

These findings establish a robust, time‐aligned dataset that bridges passive sweat sensing with validated salivary benchmarks, supporting future applications in wearable hormone classification and circadian state recognition.

**FIGURE 4:**
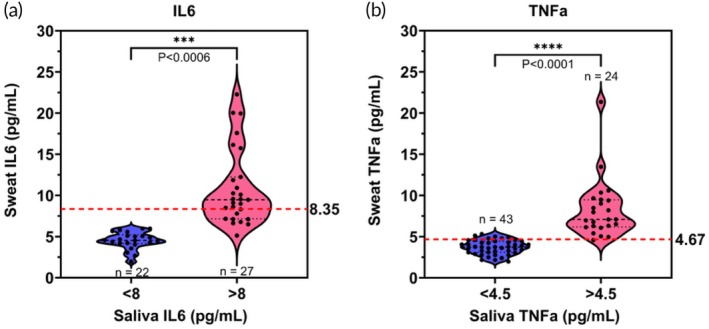
Correlation of sweat and salivary cytokine levels across validated thresholds. Violin plots illustrate sweat concentrations of IL‐6 (a) and TNF‐α (b) stratified by salivary reference bins in healthy individuals. (a) Sweat IL‐6 levels were significantly higher when salivary IL‐6 exceeded 8 pg/mL (*n* = 37); red dashed line indicates the mean sweat threshold (8.35 pg/mL); *p* = 0.0006. (b) Sweat TNF‐α levels were elevated in individuals with salivary TNF‐α above 4.5 pg/mL (*n* = 43 and 24, respectively); red dashed line denotes the mean sweat threshold (4.67 pg/mL); *p* = 0.0001. IL‐6, interleukin‐6; TNF‐α, tumor necrosis factor‐alpha.

IL‐6 and TNF‐α thresholds were also derived through sweat–saliva concordance. Sweat IL‐6 values above 8.35 pg/mL aligned with salivary levels >8 pg/mL (*p* < 0.0006) and sweat TNF‐α values exceeding 4.67 pg/mL corresponded to salivary TNF‐α >4.5 pg/mL (*p* < 0.0001). This stratification, even within a healthy cohort, provides meaningful resolution of low‐grade inflammatory variation (Figure 4a,b).

ROC curve analyses reinforced the diagnostic strength of sweat‐derived thresholds across all four biomarkers, with robust classification performance observed at both physiological extremes and across circadian windows.

For cortisol, Area Under the Curve (AUC) values ranged from 0.703 to 0.908 across salivary thresholds and time‐of‐day conditions. The sensor achieved its strongest performance in identifying low daytime salivary cortisol (<3 ng/mL) with an AUC of 0.908 (*n* = 60), and nocturnal suppression (<3 ng/mL) with an AUC of 0.829 (*n* = 123), highlighting reliable diurnal tracking. Discrimination remained strong for elevated daytime cortisol (>7 ng/mL; AUC = 0.819, *n* = 12), though performance declined slightly at intermediate ranges (e.g., <7 ng/mL, AUC = 0.703) (Figure S2).

For melatonin, the sensor demonstrated exceptional discriminatory power at low and high physiological concentrations. Daytime suppression (<10 pg/mL) was reliably classified (AUC = 0.874, *n* = 75), and nocturnal suppression yielded an AUC of 0.882 (*n* = 144). Most notably, salivary melatonin levels >40 pg/mL were detected with near‐perfect accuracy during the day (AUC = 0.984, *n* = 8) and perfectly at night (AUC = 1.00, *n* = 12). These findings underscore the sensor's ability to resolve extreme melatonin states—essential for circadian phase inference—despite limited sample sizes (Figure S3).

For inflammatory cytokines, ROC analysis of salivary comparisons yielded AUCs of 0.6656 for IL‐6 (*n* = 49) and 0.6696 for TNF‐α (*n* = 67). While moderate, these values are statistically robust in healthy cohorts where inflammatory biomarker expression is naturally low and transient. Importantly, the ROC profiles revealed that even modest sweat‐derived cytokine changes track with salivary reference levels, supporting their translational use as non‐invasive indicators of immune activation and early inflammatory signaling (Figure S4).

Together, these analyses confirm that sweat‐based sensing—when paired with validated salivary thresholds—can reliably classify hormonal and cytokine states across biological ranges and time domains, enabling real‐time physiological phenotyping.

### 3.3 Preservation and deterioration of rhythmicity using circaCompare

Passive sweat biosensing captured strong 24‐h rhythmicity across cortisol, melatonin, IL‐6, and TNF‐α, with statistically significant differences in mesor, amplitude, and peak timing across demographic and psychosocial strata. Stratification by age, gender, and self‐reported stress revealed consistent and biologically coherent signatures of circadian remodeling.

**FIGURE 5:**
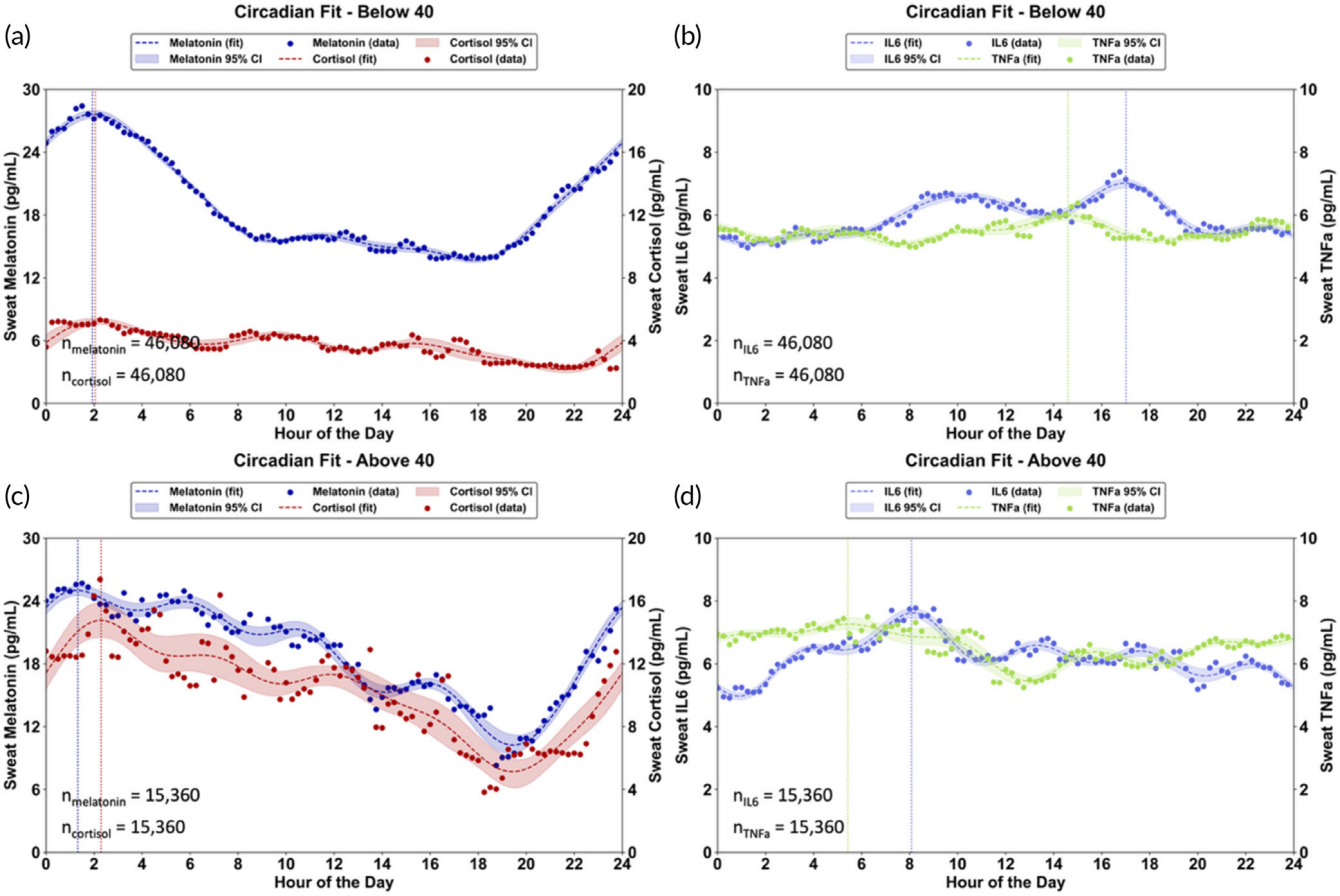
Circadian harmonic modeling of sweat melatonin, cortisol, IL‐6, and TNF‐α using circaCompare, stratified by age group (<40 and ≥ 40 years). (a, b) Younger adults (<40 years) exhibited distinct circadian rhythmicity across all biomarkers. (a) Clear phase opposition between cortisol (peak early morning) and melatonin (peak at night), along with robust amplitude. (b) Rhythmic but attenuated oscillations for IL‐6 and TNF‐α across the 24‐h cycle. (c, d) In older adults (≥40 years), (c) Blunted amplitude and reduced phase separation between melatonin and cortisol, indicating age‐related dampening of endocrine rhythmicity. (d) Flatter profiles for IL‐6 and TNF‐α, with diminished oscillatory structure compared to younger adults. IL‐6, interleukin‐6; TNF‐α, tumor necrosis factor‐alpha.

Age emerged as a major determinant of circadian organization across endocrine and immune biomarkers. Participants under 40 demonstrated well‐synchronized rhythms: cortisol peaked sharply in the early morning (6–9 a.m.), melatonin surged between 1:30 and 2:30 a.m., and both IL‐6 and TNF‐α followed low‐amplitude but distinguishable diurnal oscillations. The cortisol–melatonin amplitude difference in this group was substantial (−10.88; *p* = 0.0001), with minimal phase lag (Δ = 0.1 h; *p* = 0.89; Table S9), supporting robust hormonal counter regulation across the sleep–wake cycle (Figure 5a,b).

In contrast, individuals over 40 showed evidence of circadian drift. Although melatonin mesor remained relatively preserved, amplitude differences between cortisol and melatonin narrowed (−5.10; *p* = 0.0004), and their peak timings diverged significantly (Δ = 1.07 h; *p* = 0.0052), indicating reduced hormonal coordination (Figure 5c,d). For IL‐6 and TNF‐α, older adults exhibited flattened rhythms with broader daytime expression. Notably, TNF‐α mesors exceeded IL‐6 across sexes in this group (*p* < 0.0001), suggesting a basal shift toward pro‐inflammatory tone, a hallmark of age‐related immune remodeling.

These results highlight age‐associated blunting and desynchronization of both endocrine and cytokine circadian outputs, aligning with known declines in central pacemaker fidelity and immune regulation with aging.

Distinct differences emerged in biomarker timing and amplitude when stratified by gender.

**FIGURE 6:**
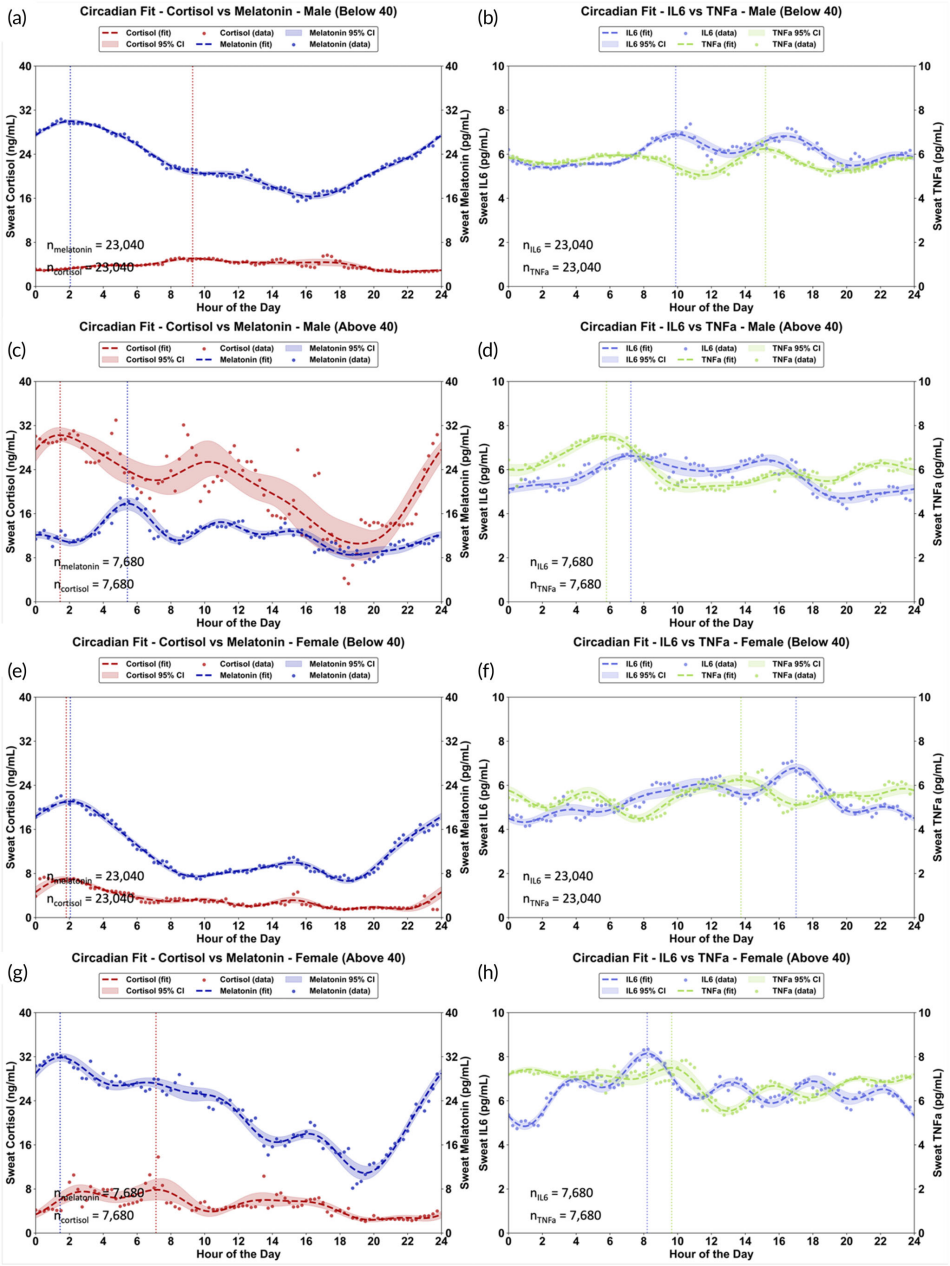
Sex‐ and age‐stratified circadian modeling of sweat‐derived melatonin, cortisol, IL‐6, and TNF‐α using harmonic regression via circaCompare. (a–d) Modeled circadian rhythms in males; (e–h) results for females, each stratified by age group (<40 and ≥40 years). (a, b) In younger males (<40 years), cortisol and melatonin (a) exhibited clear phase opposition and high amplitude with synchronized circadian periodicity (*τ* = 23.040 h), while IL‐6 and TNF‐α (b) displayed modest yet discernible rhythmic patterns. (c, d) In older males (≥40 years), cortisol and melatonin (c) showed dampened rhythmicity with reduced amplitude and phase separation (*τ* = 7.680 h), while IL‐6 and TNF‐α (d) were flattened, suggesting age‐related immune rhythm erosion. (e, f) Younger females (<40 years) showed strong phase‐opposed rhythms in cortisol and melatonin (e), again with *τ* = 23.040 h, while IL‐6 and TNF‐α (f) maintained mild rhythmicity across the 24‐h cycle. (g, h) In older females (≥40 years), both hormonal (g) and inflammatory (h) rhythms were blunted. Cortisol and melatonin amplitude declined, and IL‐6/TNF‐α periodicity contracted (*τ* = 7.680 h), indicating disrupted circadian coordination with age. IL‐6, interleukin‐6; TNF‐α, tumor necrosis factor‐alpha.

Among females under 40, cortisol and melatonin peaks were tightly aligned (ΔPeak = −0.22 h; *p* = 0.47; Table S10), with melatonin amplitude significantly greater than cortisol (Δ = −8.82; *p* = 0.0001). IL‐6 amplitude exceeded TNF‐α (Δ = 0.69; *p* = 0.0008), and peak hour separation (~3.25 h; *p* = 0.0074) suggested temporally staged immune activation (Figure 6).

By contrast, males under 40 exhibited marked cortisol–melatonin peak dissociation (Δ = 7.37 h; *p* = 0.0001), with a wider amplitude disparity (−11.21; *p* = 0.0001) and a phase‐lagging melatonin peak. Inflammatory rhythms were subtler, with only amplitude showing statistical differences between IL‐6 and TNF‐α (*p* = 0.0354), reflecting reduced immune rhythmic contrast.

In females over 40, melatonin remained dominant in amplitude (Δ = −15.08; *p* = 0.0001), but the cortisol–melatonin peak gap widened (Δ = 4.13 h; *p* = 0.0002), reflecting hormonal desynchronization potentially linked to menopausal transition. IL‐6 amplitude remained larger than TNF‐α (Δ = 1.29; *p* = 0.0001), yet peak times converged.

Males over 40 showed the inverse: cortisol amplitude exceeded melatonin (Δ = 10.99; *p* = 0.0001), with cortisol peaking nearly 4 h earlier than melatonin (*p* = 0.0232). TNF‐α surpassed IL‐6 in mesor (*p* = 0.0001), and amplitude differences reversed, suggesting a late‐life shift toward TNF‐anchored inflammatory rhythm.

These results indicate stronger hormonal coordination and immune oscillation in younger females, and more fragmented patterns in older males—with implications for personalized time‐of‐day diagnostics and interventions.

**FIGURE 7:**
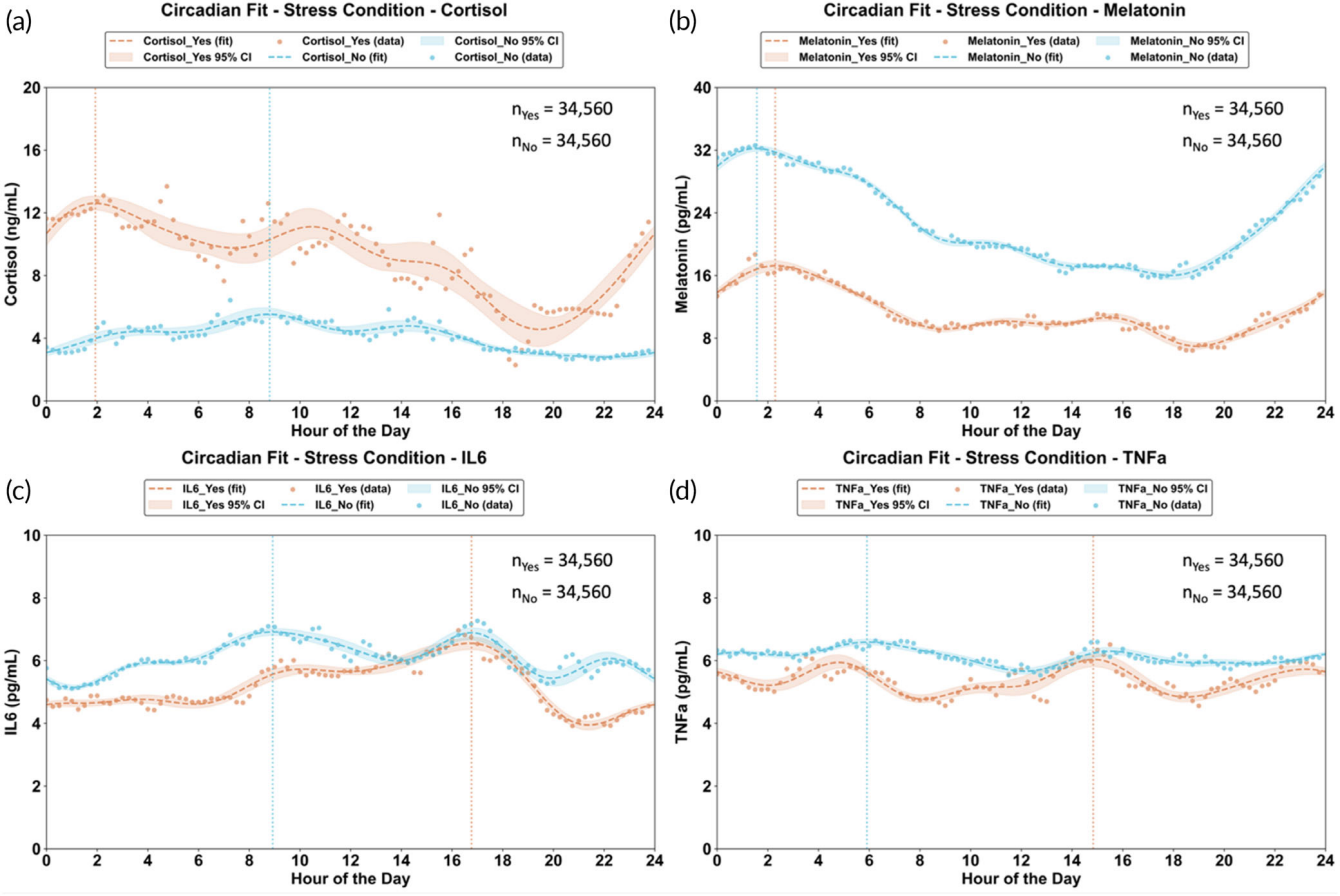
Circadian harmonic modeling of sweat biomarkers under stress conditions using circaCompare. (a–d) Twenty‐four‐hour fits of sweat cortisol (a), melatonin (b), IL‐6 (c), and TNF‐α (d) concentrations in individuals who reported they were under stress versus matched controls. IL‐6, interleukin‐6; TNF‐α, tumor necrosis factor‐alpha.

Self‐reported stress was linked to widespread alterations in the circadian profiles of all four biomarkers (Figure 7a–d). In stressed individuals, cortisol exhibited a marked elevation in its 24‐h average (mesor 9.18 vs. 4.09; *p* = 0.0001; Table S11), a significant amplification in amplitude (8.05 vs. 2.72; *p* = 0.0001), and an advance in peak timing by approximately 6.7 h (*p* = 0.0278). These changes are indicative of anticipatory activation of the HPA axis (Figure 7a).

In contrast, melatonin rhythms were suppressed under stress, with a sharp decline in mesor (>11 pg/mL lower; *p* = 0.0001), decreased amplitude (Δ = −5.91; *p* = 0.0001), and delayed peak timing (*p* = 0.0002), consistent with disrupted circadian alignment and impaired sleep‐phase signaling (Figure 7b).

For IL‐6, the stressed group demonstrated a paradoxical pattern: significantly lower mesor (*p* = 0.0001) alongside heightened amplitude (*p* = 0.0001), suggesting increased oscillatory responsiveness despite lower basal levels. TNF‐α displayed a similar response—reduced mesor and elevated amplitude (*p* < 0.01)—with large phase shifts (>6 h) that did not reach significance, potentially reflecting immune timing instability (Figure 7c,d).

Taken together, these findings indicate that psychological stress does not simply elevate biomarker levels—it disrupts the temporal architecture of hormonal and inflammatory rhythms. The emergence of compressed timing, exaggerated amplitude, and misaligned phase offers a nuanced biomarker signature of physiological strain. Importantly, these patterns were detectable through non‐invasive sweat biosensing, underscoring its value in capturing early circadian disruption and latent allostatic load.

## 4 DISCUSSION

This study introduces and validates a novel analytical framework for non‐invasive circadian and immune profiling using passive sweat biosensing, with a dual focus on^1^ biomarker stratification relative to salivary thresholds and^2^ circadian rhythmicity modeling via stratified circaCompare analysis. Together, these methods offer a comprehensive, time‐sensitive view of neuroendocrine and immune physiology—one that is scalable, interpretable, and primed for integration into real‐world wearable platforms.

Through sweat–saliva thresholding, we demonstrated that sweat concentrations of cortisol, melatonin, IL‐6, and TNF‐α can serve as reliable proxies for their salivary counterparts. The classification performance of sweat‐based thresholds—reflected in ROC AUCs consistently exceeding 0.80 for cortisol and melatonin, even in small sample bins—reinforces the signal fidelity of these analytes under both basal and biologically elevated conditions. Importantly, extreme thresholds (e.g., melatonin >40 pg/mL) achieved near‐perfect discrimination (AUC = 0.98–1.00), validating the utility of sweat sensing for detecting biologically meaningful transitions such as circadian phase onset or suppression. These findings not only support biomarker concordance but establish physiological benchmarks for device calibration.

Beyond static thresholds, the application of circaCompare harmonic modeling enabled interrogation of dynamic circadian features—mesor, amplitude, and acrophase—with respect to age, sex, and stress. This approach revealed coherent biological patterns: younger individuals exhibited synchronized high‐amplitude rhythms in melatonin and cortisol, while older adults showed amplitude dampening and phase dispersion. Younger females displayed strong melatonin–cortisol coupling and detectable immune rhythmicity, whereas older males presented with cortisol dominance and TNF‐α elevation, consistent with shifts in reproductive and inflammatory profiles.^3^, ^23^ While our stratified analysis revealed demographic patterns (age, sex, stress), individual baseline variation exists in circadian rhythmicity, the current framework establishes population‐level thresholds that provide good classification performance (AUC >0.80). However, for precision medicine applications, a brief individual calibration period (24–48 h) with paired saliva sampling could establish personalized baselines, improving sensitivity for detecting deviations from an individual's normal rhythm. Future iterations would incorporate adaptive algorithms that learn individual patterns over the first week of use.

**FIGURE 8:**
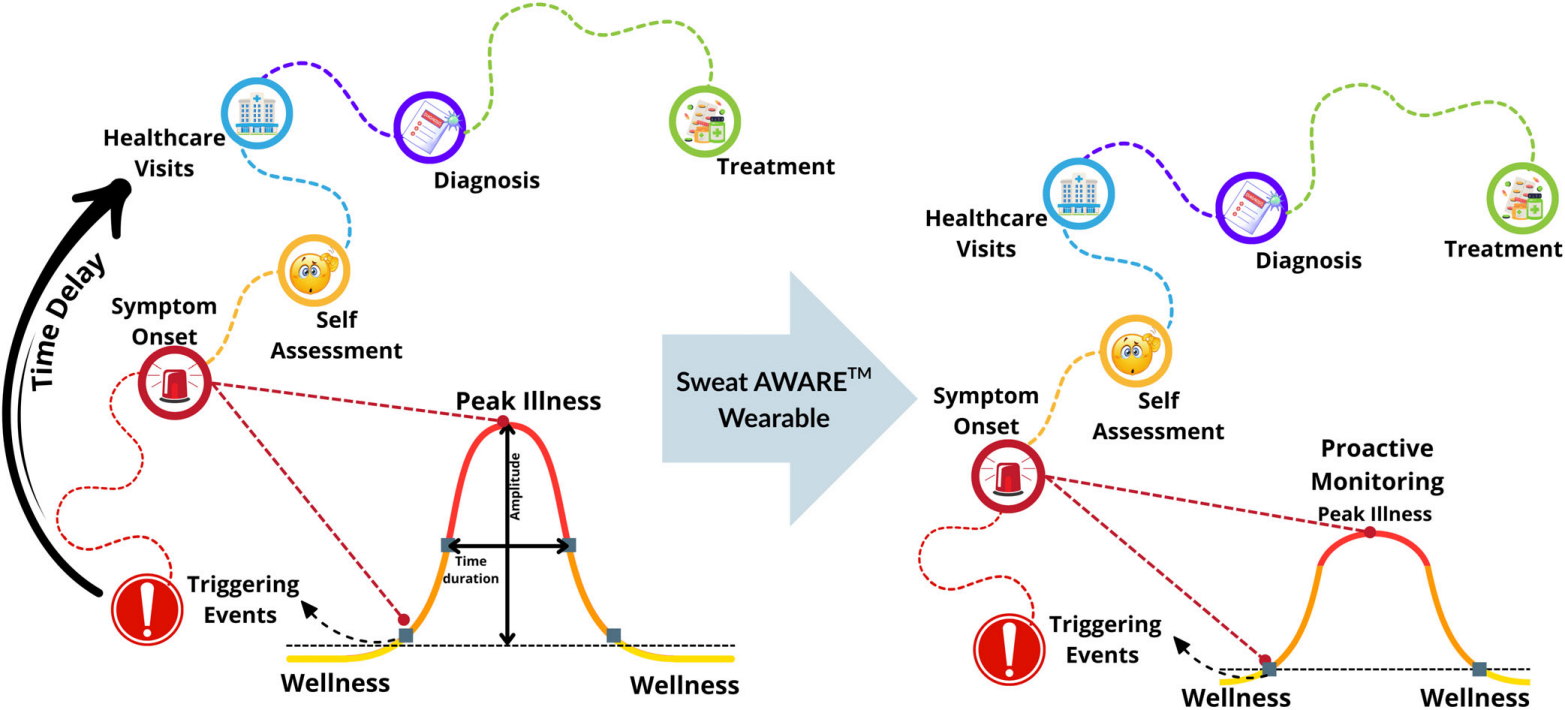
Paradigm shift enabled by continuous sweat biosensing. Left: Traditional reactive healthcare model where intervention occurs only after symptom manifestation, resulting in peak illness severity. Right: Proactive monitoring with the Sweat AWARE™ platform detects biomarker perturbations immediately following triggering events, enabling pre‐symptomatic intervention that can prevent or minimize disease flares. The temporal advantage (shown by the gap between triggering events and peak illness) represents the window of opportunity for preventive intervention.

Strikingly, self‐reported stress was linked to a decoupling of endocrine and inflammatory cycles. Cortisol amplitude tripled under stress and peaked nearly 7 h earlier (*p* = 0.0278), while melatonin amplitude declined significantly with delayed peak timing (*p* = 0.0002). Inflammatory cytokines (IL‐6 and TNF‐α) displayed reduced baseline levels (mesor) but increased amplitude (*p* < 0.01), reflecting heightened physiological volatility and immune instability. These findings align with documented stress effects on HPA axis acceleration and inflammatory tone and further reveal that sweat biosensing can detect not only concentration shifts but also deeper rhythmic reorganization of physiological networks.^24^ By coupling passive sweat analytics with time‐resolved, stratified chronobiological modeling, this study introduces a paradigm shift in real‐world circadian biomarker assessment (Figure 8). The methodological novelty lies in the dual‐validation framework—establishing physiological equivalency between sweat and salivary markers while uncovering dynamic rhythmic remodeling across biological strata. This approach transforms healthcare from the reactive model, where intervention occurs post‐symptomatically, to a proactive model where continuous monitoring enables pre‐emptive intervention during the critical window between trigger detection and symptom onset.

## 5 CONCLUSION

This study establishes a validated framework for passive circadian biosensing by integrating salivary‐aligned sweat thresholds with time‐resolved harmonic modeling of cortisol, melatonin, IL‐6, and TNF‐α. Crucially, it addresses a long‐standing challenge in chronobiology and immunology: the difficulty of capturing real‐time, biologically relevant variation in endocrine and immune markers using conventional sampling methods. Traditional serum and saliva assays are episodic, logistically constrained, and often fail to resolve dynamic rhythmic interactions—particularly in naturalistic, high‐frequency contexts where stress responses and immune changes unfold rapidly and variably.

Our findings demonstrate that sweat‐based sensing not only replicates validated salivary classifications but also uncovers nuanced rhythmic remodeling across demographic and psychological domains. This includes age‐ and sex‐associated attenuation in hormonal amplitude, and stress‐induced reorganization of phase timing and inflammatory oscillations. Such insights are rarely accessible through static or single‐timepoint sampling, underscoring the value of passive, continuous biosensing for understanding systemic coordination and breakdown.

From a translational standpoint, the framework presented here enables scalable, non‐invasive monitoring of circadian strain, stress susceptibility, and subclinical inflammation—providing a practical tool for personalized decision‐support in both clinical and everyday settings. The ability to move beyond concentration snapshots toward dynamic rhythm mapping paves the way for closed‐loop therapeutic platforms and behavior‐adaptive interventions.

By coupling passive sweat analytics with time‐resolved, stratified chronobiological modeling, this study introduces a paradigm shift in real‐world circadian biomarker assessment. The methodological novelty lies in the dual‐validation framework—establishing physiological equivalency between sweat and salivary markers while uncovering dynamic rhythmic remodeling across biological strata. Unlike prior approaches constrained by static sampling and homogeneous analysis, this strategy unlocks individualized circadian phenotypes within ecologically valid contexts. In doing so, it redefines biosensing as not merely reactive but rhythm‐aware and demographically attuned—laying the foundation for precision chrono medicine that is both accessible and anticipatory.

Future directions will focus on quantifying causal relationships between HPA axis activity and immune signaling through multi‐day, high‐resolution sweat sampling. By modeling temporal coupling and latency in stress–inflammation dynamics, we aim to develop chronotherapeutic strategies that not only detect dysregulation but proactively restore rhythmic homeostasis—a critical step toward personalized preventive care in stress‐linked disease contexts.

## AUTHOR CONTRIBUTIONS

**Annapoorna Ramasubramanya:** Writing – original draft; visualization; software; resources; methodology; formal analysis; data curation; **Preeti Singh:** Writing – original draft; visualization; validation; resources; methodology; formal analysis; data curation. **Akash Kumar:** Writing – original draft. **Kai‐Chun Lin:** Writing – review and editing; supervision; project administration. **Shalini Prasad:** Writing – review and editing; supervision; project administration; investigation; conceptualization. **Sriram Muthukumar:** Writing – review and editing; supervision; project administration; investigation; conceptualization.

## CONFLICT OF INTEREST STATEMENT

Dr. Shalini Prasad and Dr. Sriram Muthukumar have a significant interest in EnLiSense LLC, a company that may have a commercial interest in the results of this research and technology. The potential individual conflict of interest has been reviewed and managed by The University of Texas at Dallas and played no role in the study design; in the collection, analysis, and interpretation of data; in the writing of the report, or in the decision to submit the report for publication. SWEATSENSER devices and technology platforms are a proprietary of EnLiSense LLC. The authors declare that they have no known competing financial interests or personal relationships that could have appeared to influence the work reported in this paper.

## Supporting information


**Table S1.** Cortisol spike and recovery values.
**Table S2.** Melatonin spike and recovery values.
**Table S3.** IL‐6 spike and recovery values.
**Table S4.** TNF‐α spike and recovery values.
**Table S5.** Cross‐reactivity on the cortisol sensor.
**Table S6.** Cross‐reactivity on the melatonin sensor.
**Table S7.** Cross‐reactivity on IL‐6 sensor.
**Table S8.** Cross‐reactivity on TNF‐α sensor.
**Table S9.** Age‐stratified circadian parameter comparisons across endocrine and immune biomarkers. Summary of bootstrap‐derived *p*‐values and significance testing for mesor, peak hour, amplitude, and shared period estimates in matched sweat–saliva datasets. Biomarker pairs include cortisol versus melatonin (endocrine) and IL‐6 versus TNF‐α (inflammatory), stratified by participants below and above 40 years of age.
**Table S10.** Sex‐ and age‐stratified circadian comparisons across cortisol, melatonin, IL‐6, and TNF‐α biomarker pairs. Bootstrap‐derived differences and statistical significance (*p*‐values) for mesor, peak hour, amplitude, and shared period parameters across matched sweat–saliva datasets. Analyses were stratified by biomarker pair (cortisol vs. melatonin; IL‐6 vs. TNF‐α), age group (<40 vs. >40), and gender (male vs. female).
**Table S11.** Circadian biomarker differences between self‐reported stress (“Yes”) and non‐stressed (“No”) sample groups. Bootstrap‐derived comparisons of mesor, peak hour, amplitude, and rhythmic period across salivary–sweat biosensor data, stratified by participants' self‐reported stress status.
**Figure S1.** FTIR spectra of ZnO (black), DTSSP‐Ab (blue), and aptamer (green). (a) Comparison of the spectrum of ZnO, DTSSP–antibody–ZnO, and Aptamer ZnO. (b) Comparison between the only DTSSP and DTSSP‐Antibody after immobilization on ZnO. (c) Thiole (S–H) group comparison between all spectra after binding on ZnO surface.
**Figure S2.** Receiver operating characteristic (ROC) curve analysis of salivary cortisol thresholds during daytime and nighttime windows. ROC plots illustrate the classification performance of salivary cortisol bins across diurnal states.
**Figure S3.** Receiver operating characteristic (ROC) curve analysis of salivary melatonin concentration thresholds during daytime and nighttime windows. ROC plots evaluate the discriminatory performance of salivary melatonin bins for circadian classification.
**Figure S4.** Receiver operating characteristic (ROC) curve analysis of sweat–saliva cytokine equivalency for IL‐6 and TNF‐α. ROC plots compare the classification performance of inflammatory cytokine thresholds from matched sweat and saliva samples.
**Figure S5.** The SEM image depicts the porous structure of the sensor (a), and the EDS shows the confirmation of the presence of ZnO and Ag elements.

## Data Availability

The data underlying this article are proprietary and not available for sharing.
