# Supplementary material for: A wearable biosensing platform for continuous monitoring of inflammatory and metabolic biomarkers for real‐time health tracking and personalized care
Source: Bioeng Transl Med. 2026 Jan 16;11(3):e70104. doi: 10.1002/btm2.70104 (PMC13247400; doi:10.1002/btm2.70104)
Supplement: Supplementary file 1 — Table S1. Cortisol spike and recovery values. Table S2. Melatonin spike and recovery values. Table S3. IL‐6 spike and recovery values. Table S4. TNF‐α spike and recovery values. Table S5. Cross‐reactivity on the cortisol sensor. Table S6. Cross‐reactivity on the melatonin sensor. Table S7. Cross‐reactivity on IL‐6 sensor. Table S8. Cross‐reactivity on TNF‐α sensor. Table S9. Age‐stratified circadian parameter comparisons across endocrine and immune biomarkers. Summary of bootstrap‐derived p‐values and significance testing for mesor, peak hour, amplitude, and shared period estimates in matched sweat–saliva datasets. Biomarker pairs include cortisol versus melatonin (endocrine) and IL‐6 versus TNF‐α (inflammatory), stratified by participants below and above 40 years of age. Table S10. Sex‐ and age‐stratified circadian comparisons across cortisol, melatonin, IL‐6, and TNF‐α biomarker pairs. Bootstrap‐derived differences and statistical significance (p‐values) for mesor, peak hour, amplitude, and shared period parameters across matched sweat–saliva datasets. Analyses were stratified by biomarker pair (cortisol vs. melatonin; IL‐6 vs. TNF‐α), age group (<40 vs. >40), and gender (male vs. female). Table S11. Circadian biomarker differences between self‐reported stress (“Yes”) and non‐stressed (“No”) sample groups. Bootstrap‐derived comparisons of mesor, peak hour, amplitude, and rhythmic period across salivary–sweat biosensor data, stratified by participants' self‐reported stress status. Figure S1. FTIR spectra of ZnO (black), DTSSP‐Ab (blue), and aptamer (green). (a) Comparison of the spectrum of ZnO, DTSSP–antibody–ZnO, and Aptamer ZnO. (b) Comparison between the only DTSSP and DTSSP‐Antibody after immobilization on ZnO. (c) Thiole (S–H) group comparison between all spectra after binding on ZnO surface. Figure S2. Receiver operating characteristic (ROC) curve analysis of salivary cortisol thresholds during daytime and nighttime windows. ROC plots illustrate the classifi [file BTM2-11-e70104-s001.docx]

**A Wearable Biosensing Platform for Continuous Monitoring of Inflammatory and Metabolic Biomarkers for Real-Time Health Tracking and Personalized Care**

Annapoorna Ramasubramanya^1^, Preeti Singh^1^, Akash Kumar^1^, Kai-Chun Lin^1^, Shalini Prasad^1,2^, Sriram Muthukumar^2^

^1^Department of Bioengineering, University of Texas at Dallas, Richardson, TX 75080, USA

^2^EnLiSense LLC, 1813 Audubon Pondway, Allen, TX 75013, USA

Corresponding Author:

Dr. Shalini Prasad, Department of Bioengineering, University of Texas at Dallas, 800 West Campbell Rd. Mailstop: BSB11, Richardson, TX 75080-3021, e-mail: shalini.Prasad@utdallas.edu.

**
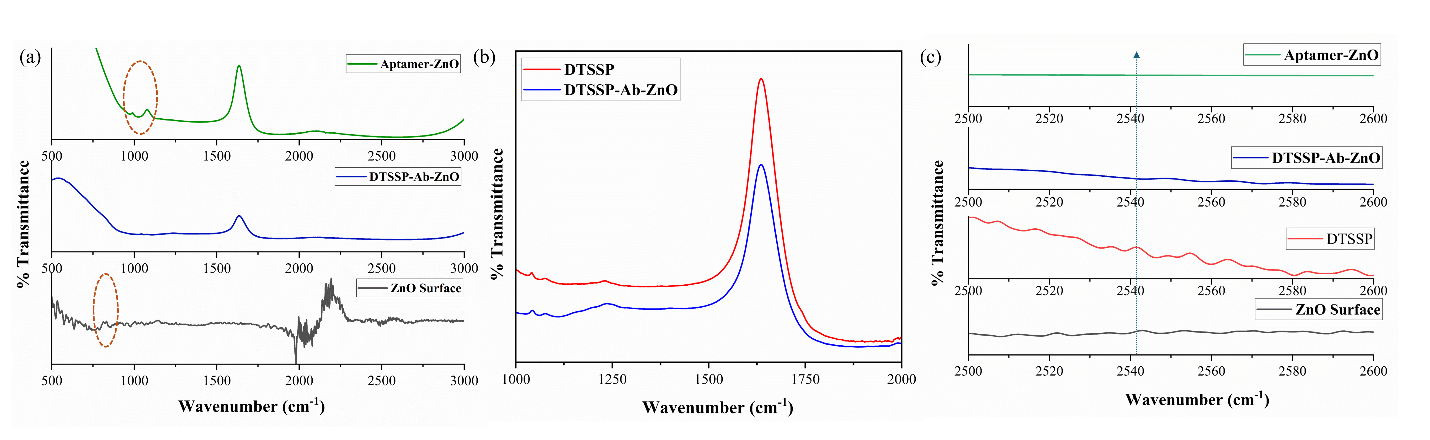
**

**Figure S1:** FTIR spectra of ZnO (black), DTSSP-Ab (blue), and aptamer (green). (a) Comparison of the spectrum of ZnO, DTSSP-Antibody-ZnO, and Aptamer ZnO. (b) Comparison between the only DTSSP and DTSSP-Antibody after immobilization on ZnO. (c) Thiole (S-H) group comparison between all spectra after binding on ZnO surface.


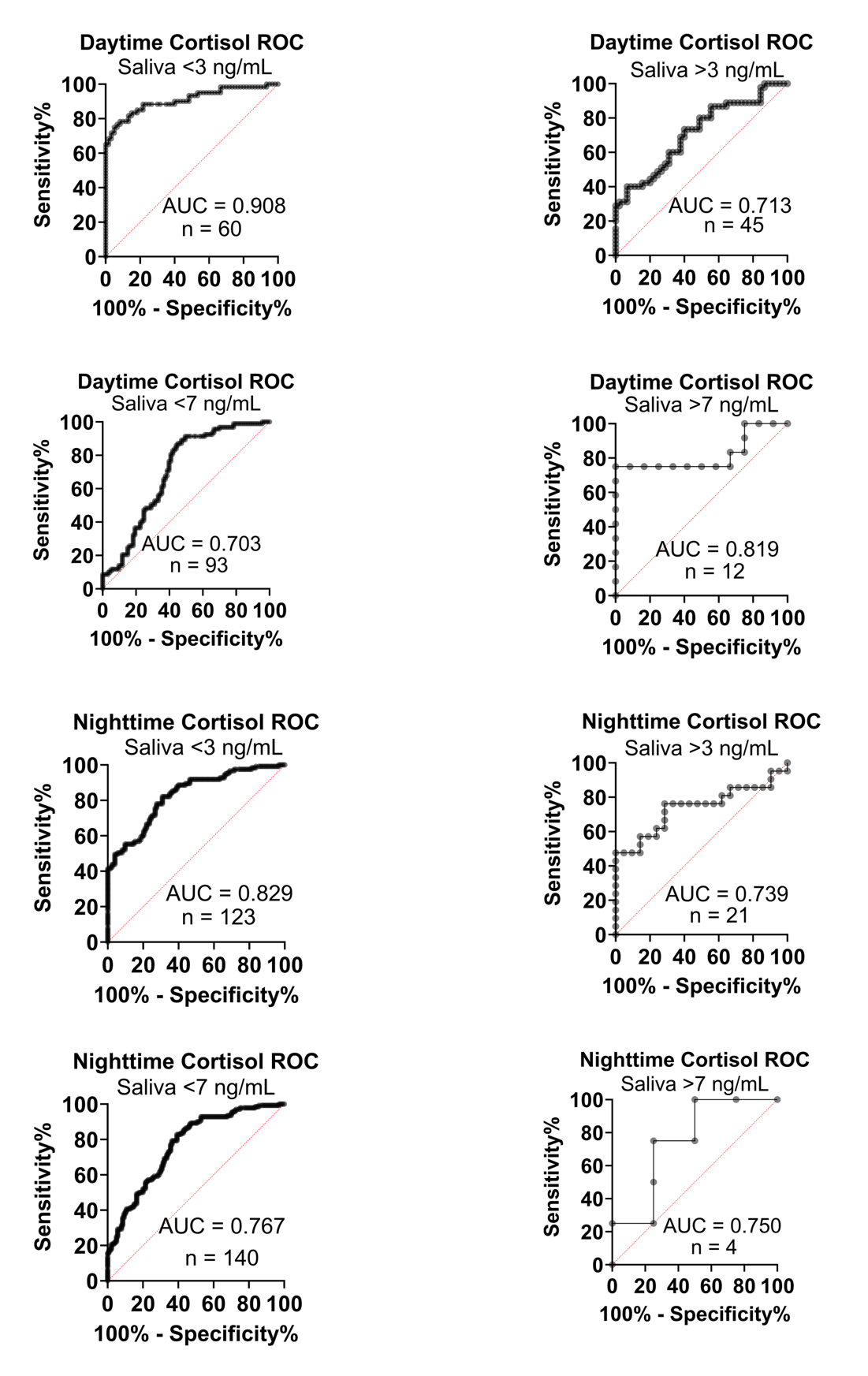


**Figure S2:** ROC curve analysis of salivary cortisol thresholds during daytime and nighttime windows. Receiver Operating Characteristic (ROC) plots illustrate the classification performance of salivary cortisol bins across diurnal states.


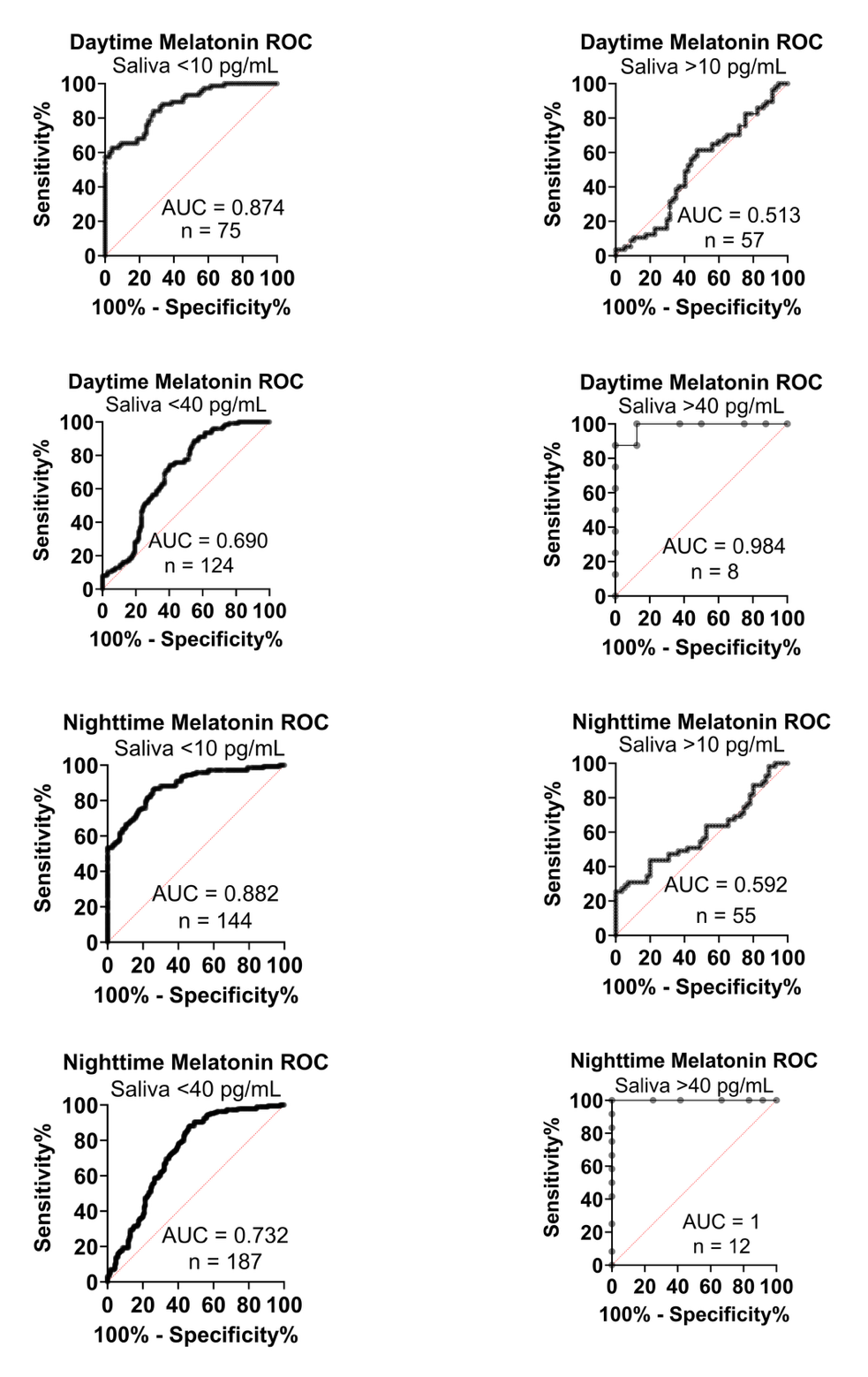


**Figure S3:** ROC curve analysis of salivary melatonin concentration thresholds during daytime and nighttime windows. Receiver Operating Characteristic (ROC) plots evaluate the discriminatory performance of salivary melatonin bins for circadian classification.


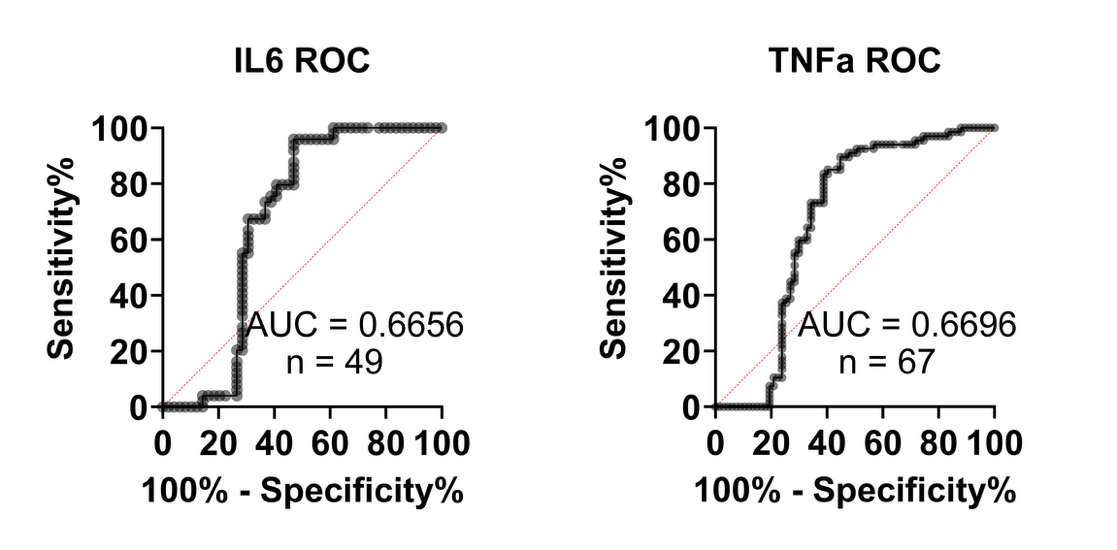


**Figure S4:** ROC curve analysis of sweat–saliva cytokine equivalency for IL6 and TNFα. Receiver Operating Characteristic (ROC) plots compare the classification performance of inflammatory cytokine thresholds from matched sweat and saliva samples.

**
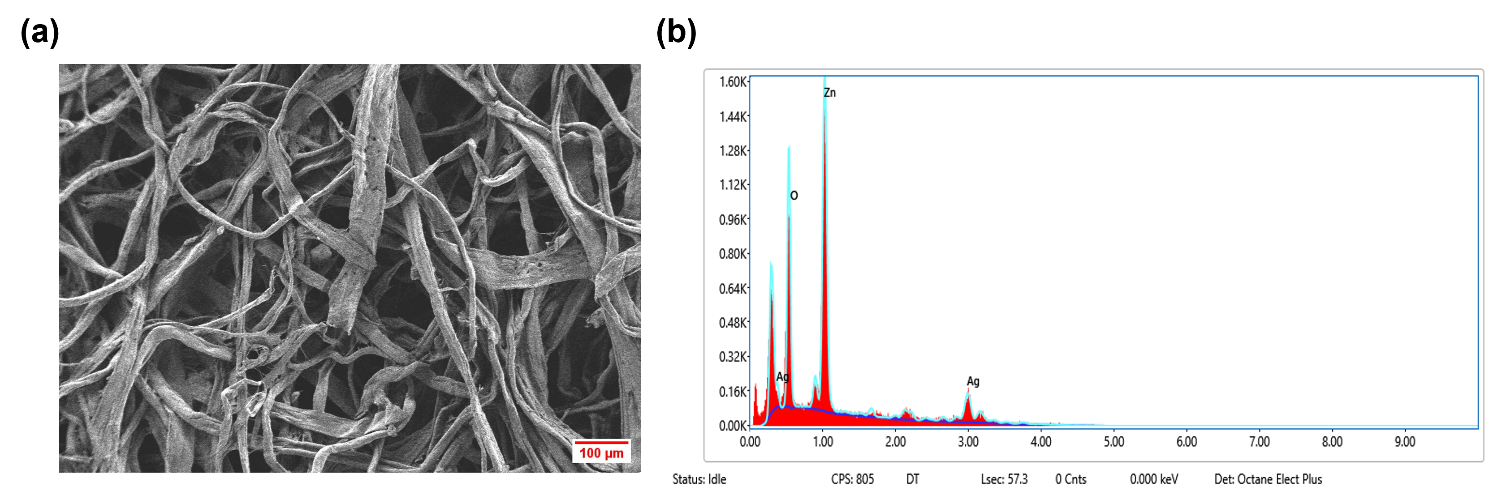
**

**Figure S5:** The SEM image depicts the porous structure of the sensor (a), and the EDS shows the confirmation of the presence of ZnO and Ag elements**.**

**Table S1:** Cortisol Spike & Recovery Values

| **Spiked concentrations**  **(ng/mL)** | **Recovered concentration**  **(ng/mL)** | **Average**  **(ng/mL)** | **Recovered Percentage (%)** |
| --- | --- | --- | --- |
| 1 | 1.373  0.800  1.578  0.778 | 1.133 | 113.276 |
| 4 | 4.352  4.068  4.274  5.175 | 4.467 | 111.682 |
| 16 | 17.608  19.641  16.621  13.817 | 16.922 | 105.761 |
| 64 | 66.723  54.919  68.864  71.056 | 65.391 | 102.173 |
| 256 | 274.004  250.452  227.359  260.336 | 253.037 | 98.843 |

**Table S2:** Melatonin Spike & Recovery Values

| **Spiked concentrations**  **(pg/mL)** | **Recovered concentration**  **(pg/mL)** | **Average**  **(pg/mL)** | **Recovered Percentage (%)** |
| --- | --- | --- | --- |
| 0.2 | 0.171  0.165  0.165  0.255 | 0.189 | 94.607 |
| 0.9 | 0.835  0.836  1.082  1.005 | 0.940 | 104.391 |
| 8.1 | 8.104  8.193  8.240  8.509 | 8.262 | 101.995 |
| 24.3 | 25.692  22.102  20.508  25.550 | 23.463 | 96.555 |
| 72.9 | 109.003  85.175  66.987  71.867 | 83.258 | 114.208 |

**Table S3:** IL-6 Spike & recovery values

| **Spiked concentrations**  **(pg/mL)** | **Recovered concentration**  **(pg/mL)** | **Average**  **(pg/mL)** | **Recovered Percentage (%)** |
| --- | --- | --- | --- |
| 1 | 1.147  1.270  0.912  1.159 | 1.122 | 112.257 |
| 4 | 3.591  3.600  4.514  4.566 | 4.068 | 101.705 |
| 16 | 16.470  16.777  16.473  17.157 | 16.719 | 104.499 |
| 64 | 62.398  79.005  51.181  69.475 | 65.515 | 102.367 |
| 256 | 205.888  298.913  258.294  266.939 | 257.509 | 100.589 |

| **Spiked concentrations**  **(ng/mL)** | **Recovered concentration**  **(ng/mL)** | **Average**  **(ng/mL)** | **Recovered Percentage (%)** |
| --- | --- | --- | --- |
| 0.1 | 0.077  0.133  0.125  0.127 | 0.116 | 115.532 |
| 10 | 9.753  14.359  10.909  8.872 | 10.973 | 109.732 |
| 50 | 51.037  47.105  53.791  53.939 | 51.468 | 102.936 |
| 100 | 129.352  95.323  103.004  102.802 | 107.620 | 107.620 |
| 200 | 192.601  233.954  224.036  214.116 | 216.177 | 108.088 |

**Table S4:** TNF-α Spike & Recovery Values

**Table S5: Cross-reactivity on the Cortisol sensor**

| **Comparable values of the Cortisol sensor** | **Concentrations used for the respective biomarkers** | **% Reactivity** |
| --- | --- | --- |
| Cortisol | 256 ng/mL | 100 % |
| IL-6 | 256 pg/mL | 9.68 % |
| TNF-ɑ | 200 pg/mL | 17.02 % |
| Melatonin | 72.9 pg/mL | 14.27 % |

**Table S6: Cross-reactivity on the Melatonin sensor**

| **Comparable values of the Melatonin ɑ sensor** | **Concentrations used for the respective biomarkers** | **% Reactivity** |
| --- | --- | --- |
| Melatonin | 72.9 pg/mL | 100 % |
| IL-6 | 256 pg/mL | 4.30 % |
| TNF-ɑ | 200 pg/mL | 5.24 % |
| Cortisol | 256 ng/mL | 1.65 % |

**Table S7: Cross-reactivity on IL-6 sensor**

| **Comparable values of the IL-6 sensor (pg/mL)** | **Concentrations used for the respective biomarkers** | **% Reactivity** |
| --- | --- | --- |
| IL-6 | 256 pg/mL | 100 % |
| TNF-ɑ | 200 pg/mL | 0.66 % |
| Cortisol | 256 ng/mL | 3.45 % |
| Melatonin | 72.9 pg/mL | 3.54 % |

**Table S8: Cross-reactivity on TNF-ɑ sensor**

| **Comparable values of the TNF-ɑ sensor (pg/mL)** | **Concentrations used for the respective biomarkers** | **% Reactivity** |
| --- | --- | --- |
| TNF-ɑ | 200 pg/mL | 100 % |
| IL-6 | 256 pg/mL | 17.72 % |
| Cortisol | 256 ng/mL | 6.14 % |
| Melatonin | 72.9 pg/mL | 5.77 % |

**Table S9:** Age-stratified circadian parameter comparisons across endocrine and immune biomarkers. Summary of bootstrap-derived p-values and significance testing for mesor, peak hour, amplitude, and shared period estimates in matched sweat–saliva datasets. Biomarker pairs include Cortisol vs. Melatonin (endocrine) and IL6 vs. TNFα (inflammatory), stratified by participants below and above 40 years of age.

| **Metric** | **Value** | **p-value (bootstrap)** | **Significant (p<0.05, boot)** | **Biomarker_Pair** | **Age_Group** |
| --- | --- | --- | --- | --- | --- |
| Cortisol mesor | 3.711712834 |  |  | Cortisol_vs_Melatonin | Below 40 |
| Melatonin mesor | 18.90295842 |  |  | Cortisol_vs_Melatonin | Below 40 |
| Mesor: Cortisol vs Melatonin | -15.19143321 | 0.0001 | Yes | Cortisol_vs_Melatonin | Below 40 |
| Cortisol peak hour | 2.050251256 |  |  | Cortisol_vs_Melatonin | Below 40 |
| Melatonin peak hour | 1.929648241 |  |  | Cortisol_vs_Melatonin | Below 40 |
| Peak Hour: Cortisol vs Melatonin | 0.097965829 | 0.8926 | No | Cortisol_vs_Melatonin | Below 40 |
| Amplitude (Cortisol) | 3.059691804 |  |  | Cortisol_vs_Melatonin | Below 40 |
| Amplitude (Melatonin) | 13.95800371 |  |  | Cortisol_vs_Melatonin | Below 40 |
| Amplitude: Cortisol vs Melatonin | -10.8776956 | 0.0001 | Yes | Cortisol_vs_Melatonin | Below 40 |
| Shared period estimate | 24 |  |  | Cortisol_vs_Melatonin | Below 40 |
| Cortisol mesor | 10.30978227 |  |  | Cortisol_vs_Melatonin | Above 40 |
| Melatonin mesor | 18.93629722 |  |  | Cortisol_vs_Melatonin | Above 40 |
| Mesor: Cortisol vs Melatonin | -8.624459042 | 0.0001 | Yes | Cortisol_vs_Melatonin | Above 40 |
| Cortisol peak hour | 2.291457286 |  |  | Cortisol_vs_Melatonin | Above 40 |
| Melatonin peak hour | 1.326633166 |  |  | Cortisol_vs_Melatonin | Above 40 |
| Peak Hour: Cortisol vs Melatonin | 1.071907538 | 0.0052 | Yes | Cortisol_vs_Melatonin | Above 40 |
| Amplitude (Cortisol) | 9.626806247 |  |  | Cortisol_vs_Melatonin | Above 40 |
| Amplitude (Melatonin) | 14.7890473 |  |  | Cortisol_vs_Melatonin | Above 40 |
| Amplitude: Cortisol vs Melatonin | -5.104010797 | 0.0004 | Yes | Cortisol_vs_Melatonin | Above 40 |
| Shared period estimate | 24 |  |  | Cortisol_vs_Melatonin | Above 40 |
| IL6 mesor | 5.913417208 |  |  | IL6_vs_TNFa | Below 40 |
| TNFa mesor | 5.456334994 |  |  | IL6_vs_TNFa | Below 40 |
| Mesor: IL6 vs TNFa | 0.457543234 | 0.0001 | Yes | IL6_vs_TNFa | Below 40 |
| IL6 peak hour | 17.00502513 |  |  | IL6_vs_TNFa | Below 40 |
| TNFa peak hour | 14.59296482 |  |  | IL6_vs_TNFa | Below 40 |
| Peak Hour: IL6 vs TNFa | 2.401953769 | 0.0044 | Yes | IL6_vs_TNFa | Below 40 |
| Amplitude (IL6) | 1.918641819 |  |  | IL6_vs_TNFa | Below 40 |
| Amplitude (TNFa) | 0.916344069 |  |  | IL6_vs_TNFa | Below 40 |
| Amplitude: IL6 vs TNFa | 0.99930421 | 0.0001 | Yes | IL6_vs_TNFa | Below 40 |
| Shared period estimate | 24 |  |  | IL6_vs_TNFa | Below 40 |
| IL6 mesor | 6.224803786 |  |  | IL6_vs_TNFa | Above 40 |
| TNFa mesor | 6.554699698 |  |  | IL6_vs_TNFa | Above 40 |
| Mesor: IL6 vs TNFa | -0.330024219 | 0.0001 | Yes | IL6_vs_TNFa | Above 40 |
| IL6 peak hour | 8.08040201 |  |  | IL6_vs_TNFa | Above 40 |
| TNFa peak hour | 5.427135678 |  |  | IL6_vs_TNFa | Above 40 |
| Peak Hour: IL6 vs TNFa | 2.589057286 | 0.0416 | Yes | IL6_vs_TNFa | Above 40 |
| Amplitude (IL6) | 2.645601097 |  |  | IL6_vs_TNFa | Above 40 |
| Amplitude (TNFa) | 1.805863763 |  |  | IL6_vs_TNFa | Above 40 |
| Amplitude: IL6 vs TNFa | 0.827325694 | 0.0002 | Yes | IL6_vs_TNFa | Above 40 |
| Shared period estimate | 24 |  |  | IL6_vs_TNFa | Above 40 |

**Table S10:** Sex- and age-stratified circadian comparisons across cortisol, melatonin, IL6, and TNFα biomarker pairs. Bootstrap-derived differences and statistical significance (p-values) for mesor, peak hour, amplitude, and shared period parameters across matched sweat–saliva datasets. Analyses were stratified by biomarker pair (Cortisol vs. Melatonin; IL6 vs. TNFα), age group (<40 vs. >40), and gender (male vs. female).

| **Metric** | **Value** | **p-value (bootstrap)** | **Biomarker_Pair** | **Age_Group** | **Gender** |
| --- | --- | --- | --- | --- | --- |
| Cortisol mesor | 3.868247254 |  | Cortisol_vs_Melatonin | Below 40 | Male |
| Melatonin mesor | 22.52869601 |  | Cortisol_vs_Melatonin | Below 40 | Male |
| Mesor Difference | -24.48835341 | 0.0001 | Cortisol_vs_Melatonin | Below 40 | Male |
| Cortisol peak hour | 9.286432161 |  | Cortisol_vs_Melatonin | Below 40 | Male |
| Melatonin peak hour | 2.050251256 |  | Cortisol_vs_Melatonin | Below 40 | Male |
| Peak Hour Difference | 7.367252261 | 0.0001 | Cortisol_vs_Melatonin | Below 40 | Male |
| Amplitude (Cortisol) | 2.436712966 |  | Cortisol_vs_Melatonin | Below 40 | Male |
| Amplitude (Melatonin) | 13.64985801 |  | Cortisol_vs_Melatonin | Below 40 | Male |
| Amplitude Difference | -11.21259147 | 0.0001 | Cortisol_vs_Melatonin | Below 40 | Male |
| Shared period estimate | 24 |  | Cortisol_vs_Melatonin | Below 40 | Male |
| Cortisol mesor | 3.278270839 |  | Cortisol_vs_Melatonin | Below 40 | Female |
| Melatonin mesor | 12.06225352 |  | Cortisol_vs_Melatonin | Below 40 | Female |
| Mesor Difference | -13.75298024 | 0.0001 | Cortisol_vs_Melatonin | Below 40 | Female |
| Cortisol peak hour | 1.809045226 |  | Cortisol_vs_Melatonin | Below 40 | Female |
| Melatonin peak hour | 2.050251256 |  | Cortisol_vs_Melatonin | Below 40 | Female |
| Peak Hour Difference | -0.222343719 | 0.4666 | Cortisol_vs_Melatonin | Below 40 | Female |
| Amplitude (Cortisol) | 5.54336158 |  | Cortisol_vs_Melatonin | Below 40 | Female |
| Amplitude (Melatonin) | 14.43277907 |  | Cortisol_vs_Melatonin | Below 40 | Female |
| Amplitude Difference | -8.816846885 | 0.0001 | Cortisol_vs_Melatonin | Below 40 | Female |
| Shared period estimate | 24 |  | Cortisol_vs_Melatonin | Below 40 | Female |
| Cortisol mesor | 21.10868359 |  | Cortisol_vs_Melatonin | Above 40 | Male |
| Melatonin mesor | 12.14137891 |  | Cortisol_vs_Melatonin | Above 40 | Male |
| Mesor Difference | 15.48315019 | 0.0001 | Cortisol_vs_Melatonin | Above 40 | Male |
| Cortisol peak hour | 1.447236181 |  | Cortisol_vs_Melatonin | Above 40 | Male |
| Melatonin peak hour | 5.427135678 |  | Cortisol_vs_Melatonin | Above 40 | Male |
| Peak Hour Difference | -3.861394975 | 0.0232 | Cortisol_vs_Melatonin | Above 40 | Male |
| Amplitude (Cortisol) | 19.69005224 |  | Cortisol_vs_Melatonin | Above 40 | Male |
| Amplitude (Melatonin) | 9.321791764 |  | Cortisol_vs_Melatonin | Above 40 | Male |
| Amplitude Difference | 10.98811396 | 0.0001 | Cortisol_vs_Melatonin | Above 40 | Male |
| Shared period estimate | 24 |  | Cortisol_vs_Melatonin | Above 40 | Male |
| Cortisol mesor | 5.122517506 |  | Cortisol_vs_Melatonin | Above 40 | Female |
| Melatonin mesor | 22.29593519 |  | Cortisol_vs_Melatonin | Above 40 | Female |
| Mesor Difference | -25.61275984 | 0.0001 | Cortisol_vs_Melatonin | Above 40 | Female |
| Cortisol peak hour | 7.115577889 |  | Cortisol_vs_Melatonin | Above 40 | Female |
| Melatonin peak hour | 1.447236181 |  | Cortisol_vs_Melatonin | Above 40 | Female |
| Peak Hour Difference | 4.134512563 | 0.0002 | Cortisol_vs_Melatonin | Above 40 | Female |
| Amplitude (Cortisol) | 5.46865676 |  | Cortisol_vs_Melatonin | Above 40 | Female |
| Amplitude (Melatonin) | 20.9732625 |  | Cortisol_vs_Melatonin | Above 40 | Female |
| Amplitude Difference | -15.08212761 | 0.0001 | Cortisol_vs_Melatonin | Above 40 | Female |
| Shared period estimate | 24 |  | Cortisol_vs_Melatonin | Above 40 | Female |
| IL6 mesor | 6.037314236 |  | IL6_vs_TNFa | Below 40 | Male |
| TNFa mesor | 5.647859632 |  | IL6_vs_TNFa | Below 40 | Male |
| Mesor Difference | 0.026585881 | 0.6958 | IL6_vs_TNFa | Below 40 | Male |
| IL6 peak hour | 9.889447236 |  | IL6_vs_TNFa | Below 40 | Male |
| TNFa peak hour | 15.1959799 |  | IL6_vs_TNFa | Below 40 | Male |
| Peak Hour Difference | -3.964160804 | 0.3978 | IL6_vs_TNFa | Below 40 | Male |
| Amplitude (IL6) | 1.517021388 |  | IL6_vs_TNFa | Below 40 | Male |
| Amplitude (TNFa) | 1.181753225 |  | IL6_vs_TNFa | Below 40 | Male |
| Amplitude Difference | 0.357951797 | 0.0354 | IL6_vs_TNFa | Below 40 | Male |
| Shared period estimate | 24 |  | IL6_vs_TNFa | Below 40 | Male |
| IL6 mesor | 5.376206697 |  | IL6_vs_TNFa | Below 40 | Female |
| TNFa mesor | 5.464045023 |  | IL6_vs_TNFa | Below 40 | Female |
| Mesor Difference | -1.258770522 | 0.0001 | IL6_vs_TNFa | Below 40 | Female |
| IL6 peak hour | 17.00502513 |  | IL6_vs_TNFa | Below 40 | Female |
| TNFa peak hour | 13.74874372 |  | IL6_vs_TNFa | Below 40 | Female |
| Peak Hour Difference | 3.253628141 | 0.0074 | IL6_vs_TNFa | Below 40 | Female |
| Amplitude (IL6) | 2.453499107 |  | IL6_vs_TNFa | Below 40 | Female |
| Amplitude (TNFa) | 1.747758461 |  | IL6_vs_TNFa | Below 40 | Female |
| Amplitude Difference | 0.693578907 | 0.0008 | IL6_vs_TNFa | Below 40 | Female |
| Shared period estimate | 24 |  | IL6_vs_TNFa | Below 40 | Female |
| IL6 mesor | 5.693347222 |  | IL6_vs_TNFa | Above 40 | Male |
| TNFa mesor | 6.041789063 |  | IL6_vs_TNFa | Above 40 | Male |
| Mesor Difference | -0.890935665 | 0.0001 | IL6_vs_TNFa | Above 40 | Male |
| IL6 peak hour | 7.236180905 |  | IL6_vs_TNFa | Above 40 | Male |
| TNFa peak hour | 5.788944724 |  | IL6_vs_TNFa | Above 40 | Male |
| Peak Hour Difference | 2.117439196 | 0.0001 | IL6_vs_TNFa | Above 40 | Male |
| Amplitude (IL6) | 1.92492871 |  | IL6_vs_TNFa | Above 40 | Male |
| Amplitude (TNFa) | 2.313576273 |  | IL6_vs_TNFa | Above 40 | Male |
| Amplitude Difference | -0.390025086 | 0.0158 | IL6_vs_TNFa | Above 40 | Male |
| Shared period estimate | 24 |  | IL6_vs_TNFa | Above 40 | Male |
| IL6 mesor | 6.484792523 |  | IL6_vs_TNFa | Above 40 | Female |
| TNFa mesor | 6.803684462 |  | IL6_vs_TNFa | Above 40 | Female |
| Mesor Difference | -1.873189555 | 0.0001 | IL6_vs_TNFa | Above 40 | Female |
| IL6 peak hour | 8.201005025 |  | IL6_vs_TNFa | Above 40 | Female |
| TNFa peak hour | 9.648241206 |  | IL6_vs_TNFa | Above 40 | Female |
| Peak Hour Difference | 1.421125628 | 0.6914 | IL6_vs_TNFa | Above 40 | Female |
| Amplitude (IL6) | 3.30271364 |  | IL6_vs_TNFa | Above 40 | Female |
| Amplitude (TNFa) | 1.957184393 |  | IL6_vs_TNFa | Above 40 | Female |
| Amplitude Difference | 1.290631038 | 0.0001 | IL6_vs_TNFa | Above 40 | Female |
| Shared period estimate | 24 |  | IL6_vs_TNFa | Above 40 | Female |

**Table S11:** Circadian biomarker differences between self-reported stress (“Yes”) and non-stressed (“No”) sample groups. Bootstrap-derived comparisons of mesor, peak hour, amplitude, and rhythmic period across salivary–sweat biosensor data, stratified by participants' self-reported stress status.

| **Metric** | **Value** | **p-value (bootstrap)** | **Significant (p<0.05, boot)** | **Biomarker** |
| --- | --- | --- | --- | --- |
| Cortisol_Yes mesor | 9.180661727 |  |  | Cortisol |
| Cortisol_No mesor | 4.088688602 |  |  | Cortisol |
| Mesor: Cortisol_Yes vs Cortisol_No | 5.090515724 | 0.0001 | Yes | Cortisol |
| Cortisol_Yes peak hour | 1.929648241 |  |  | Cortisol |
| Cortisol_No peak hour | 8.804020101 |  |  | Cortisol |
| Peak Hour: Cortisol_Yes vs Cortisol_No | -6.700293467 | 0.0278 | Yes | Cortisol |
| Amplitude (Cortisol_Yes) | 8.046439109 |  |  | Cortisol |
| Amplitude (Cortisol_No) | 2.719729718 |  |  | Cortisol |
| Amplitude: Cortisol_Yes vs Cortisol_No | 5.407493477 | 0.0001 | Yes | Cortisol |
| Shared period estimate | 24 |  |  | Cortisol |
| Melatonin_Yes mesor | 11.25172687 |  |  | Melatonin |
| Melatonin_No mesor | 22.77201231 |  |  | Melatonin |
| Mesor: Melatonin_Yes vs Melatonin_No | -11.51876035 | 0.0001 | Yes | Melatonin |
| Melatonin_Yes peak hour | 2.291457286 |  |  | Melatonin |
| Melatonin_No peak hour | 1.567839196 |  |  | Melatonin |
| Peak Hour: Melatonin_Yes vs Melatonin_No | 0.817628141 | 0.0002 | Yes | Melatonin |
| Amplitude (Melatonin_Yes) | 10.27755731 |  |  | Melatonin |
| Amplitude (Melatonin_No) | 16.20016327 |  |  | Melatonin |
| Amplitude: Melatonin_Yes vs Melatonin_No | -5.91326338 | 0.0001 | Yes | Melatonin |
| Shared period estimate | 24 |  |  | Melatonin |
| IL6_Yes mesor | 5.197252636 |  |  | IL6 |
| IL6_No mesor | 6.117797798 |  |  | IL6 |
| Mesor: IL6_Yes vs IL6_No | -0.920908569 | 0.0001 | Yes | IL6 |
| IL6_Yes peak hour | 16.7638191 |  |  | IL6 |
| IL6_No peak hour | 8.924623116 |  |  | IL6 |
| Peak Hour: IL6_Yes vs IL6_No | 4.369133668 | 0.6318 | No | IL6 |
| Amplitude (IL6_Yes) | 2.610631124 |  |  | IL6 |
| Amplitude (IL6_No) | 1.777253572 |  |  | IL6 |
| Amplitude: IL6_Yes vs IL6_No | 0.802640111 | 0.0001 | Yes | IL6 |
| Shared period estimate | 24 |  |  | IL6 |
| TNFa_Yes mesor | 5.372632908 |  |  | TNFa |
| TNFa_No mesor | 6.121766733 |  |  | TNFa |
| Mesor: TNFa_Yes vs TNFa_No | -0.749483729 | 0.0001 | Yes | TNFa |
| TNFa_Yes peak hour | 14.83417085 |  |  | TNFa |
| TNFa_No peak hour | 5.909547739 |  |  | TNFa |
| Peak Hour: TNFa_Yes vs TNFa_No | 6.64798794 | 0.443 | No | TNFa |
| Amplitude (TNFa_Yes) | 1.260287639 |  |  | TNFa |
| Amplitude (TNFa_No) | 0.925891613 |  |  | TNFa |
| Amplitude: TNFa_Yes vs TNFa_No | 0.380140396 | 0.0032 | Yes | TNFa |
| Shared period estimate | 24 |  |  | TNFa |


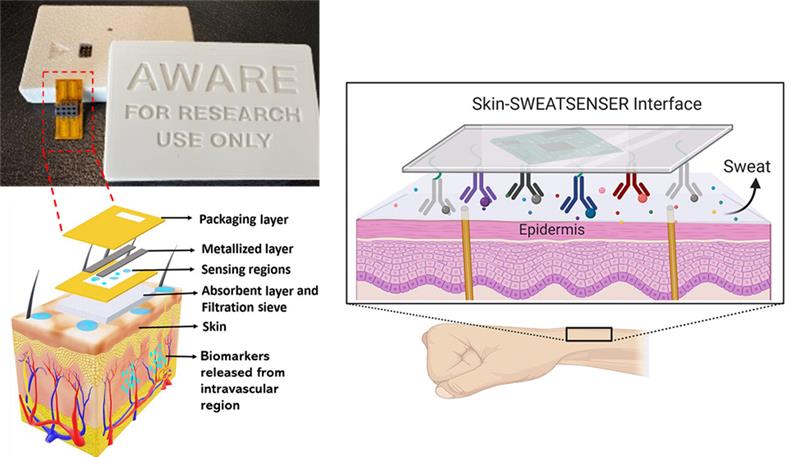


**Figure S5:** Illustration of the AWARE–SWEATSENSER platform showing the wearable electrochemical module, the multilayer SWEATSENSER strip architecture, and the skin–sensor interface. The device integrates metallized contacts, functionalized sensing regions, and an absorbent filtration layer to continuously sample passive eccrine sweat, enabling selective capture of biomarkers as they diffuse from the intravascular space to the skin surface.
